# Convergent evolution involving dimeric and trimeric dUTPases in pathogenicity island mobilization

**DOI:** 10.1371/journal.ppat.1006581

**Published:** 2017-09-11

**Authors:** Jorge Donderis, Janine Bowring, Elisa Maiques, J. Rafael Ciges-Tomas, Christian Alite, Iltyar Mehmedov, María Angeles Tormo-Mas, José R. Penadés, Alberto Marina

**Affiliations:** 1 Instituto de Biomedicina de Valencia (IBV-CSIC) and CIBER de Enfermedades Raras (CIBERER), Valencia, Spain; 2 Institute of Infection, Immunity and Inflammation, College of Medical, Veterinary and Life Sciences, University of Glasgow, Glasgow, United Kingdom; 3 Departamento de Ciencias Biomédicas, Universidad CEU Cardenal Herrera, Moncada, Valencia, Spain; University of Tubingen, GERMANY

## Abstract

The dUTPase (Dut) enzymes, encoded by almost all free-living organisms and some viruses, prevent the misincorporation of uracil into DNA. We previously proposed that trimeric Duts are regulatory proteins involved in different cellular processes; including the phage-mediated transfer of the *Staphylococcus aureus* pathogenicity island SaPIbov1. Recently, it has been shown that the structurally unrelated dimeric Dut encoded by phage ϕNM1 is similarly able to mobilize SaPIbov1, suggesting dimeric Duts could also be regulatory proteins. How this is accomplished remains unsolved. Here, using *in vivo*, biochemical and structural approaches, we provide insights into the signaling mechanism used by the dimeric Duts to induce the SaPIbov1 cycle. As reported for the trimeric Duts, dimeric Duts contain an extremely variable region, here named domain VI, which is involved in the regulatory capacity of these enzymes. Remarkably, our results also show that the dimeric Dut signaling mechanism is modulated by dUTP, as with the trimeric Duts. Overall, our results demonstrate that although unrelated both in sequence and structure, dimeric and trimeric Duts control SaPI transfer by analogous mechanisms, representing a fascinating example of convergent evolution. This conserved mode of action highlights the biological significance of Duts as regulatory molecules.

## Author summary

Understanding bacterial horizontal gene transfer is vital for establishing how toxins and antibiotic resistance genes are disseminated. Bacteriophage and pathogenicity islands make up key components of the wider horizontal transfer map. SaPIs are clinically relevant pathogenicity islands, residing passively in the host chromosome under control of the SaPI master repressor, Stl. We have recently described that the trimeric phage-encoded dUTPases (Dut) act as anti-repressors for a subset of SaPIs, by interacting with an identical encoded Stl. Some *Staphylococcus aureus* phages encode dimeric instead of trimeric Duts, which are completely unrelated at both sequence and structural levels. Recent work involved the dimeric Dut from *S*. *aureus* phage ϕNM1 in the mobilization of SaPIbov1, which is also induced by trimeric Duts, suggesting a similar mechanism of SaPIbov1 induction. Here, our analysis of SaPIbov1 induction reveals the existence of several allelic forms of dimeric Dut, with varying capacity for SaPI de-repression. The three-dimensional structure of one de-repressing dimeric Dut shows a simplified version of these enzymes, with functional particularities compared to any previously characterised dimeric Dut. Remarkably for unrelated proteins, our results indicate the de-repression mechanism used by dimeric and trimeric Duts is conceptually identical, involving dUTP as a second messenger turning off Stl-Dut interaction. Our findings show the significance of Duts as signalling molecules, representing a fascinating example convergent evolution in action.

## Introduction

The staphylococcal pathogenicity islands (SaPIs) are virus satellites that carry and disseminate virulence genes in *Staphylococcus aureus*. They reside passively in the host chromosome under the control of Stl, a global SaPI-encoded repressor. Following infection by a helper phage, they excise, replicate, and are packaged in phage-like particles composed of phage virion proteins, leading to very high frequencies of both inter- and intrageneric transfer [1]. The SaPI cycle is induced by a specific phage-encoded protein, which binds to the SaPI-encoded repressor, Stl, to act as an antirepressor [2]. Different SaPIs encode different Stl repressors, so each requires a specific phage protein for its de-repression. Thus, while the inducer for SaPI1 is the phage-encoded Sri protein, the inducer for SaPIbov2 is the 80α ORF15 [2]. Interestingly, it was initially demonstrated that the trimeric phage-encoded dUTPase (Dut) proteins are the de-repressor proteins for a subset of SaPIs, including SaPIbov1, SaPIbov5 and SaPIov1, all of which encode the same Stl repressor [2, 3]. SaPI de-repression by phage trimeric Duts depends on the catalytically conserved motifs III, IV and V, as well as on the presence of a non-conserved specific motif in these phage Duts, that we have called motif VI. The variability of motif VI accounts for the existence of a high number of trimeric Dut allelic variants with different affinities for the SaPI encoded Stl repressor [2, 4], this being a mechanism used by the phages to avoid SaPI induction [5]. Interestingly, SaPI de-repression by the trimeric Duts also involves dUTP as the second messenger molecule in a conceptual parallelism with the signaling mechanism of eukaryotic G proteins [3, 6]. In this case, however, the dUTP interferes with the Dut-mediated induction of the SaPIbov1 cycle [6, 7].

In a parallel study we noticed that some *S*. *aureus* phages encode dimeric instead of trimeric Duts [5, 8, 9]. We speculated that substitution of trimeric by dimeric Duts could be a mechanism used by the phages to avoid SaPI induction, which is detrimental for the phage cycle [10], while maintaining dUTPase activity. Remarkably, in a recent work, Hill and Dokland involved one of these predicted dimeric Duts, from phage ϕNM1, in the mobilization of SaPIbov1 [11]. This was a striking observation since previous studies using other model organisms had demonstrated that dimeric and trimeric Duts are completely unrelated at both sequence and structural levels [9, 12, 13]. Trimeric Duts are the most abundant in nature and are found from viruses to humans [12, 13]. These Duts have been extensively characterized at a structural level, including characterization of Duts codified by the staphylococcal 80α and ϕ11 phages, showing that their fold is composed of β-sheets [3, 7, 13]. Meanwhile, dimeric Duts represent a reduced group only present in trypanosomatides, some bacteria and some bacteriophages [9]. Only four structures of dimeric Duts have been reported, those from *Trypanosoma cruzi* and *Trypanosoma brucei*, *Campylobacter jejuni* and *Leishmania major* [8, 14–16], confirming that this type of Dut presents an all-helical structure. Furthermore, at a sequence level the five catalytic motifs present in the dimeric Duts are completely different to those conforming the active site in the trimeric Duts [8, 13]. These differences account for the differences in nucleotide recognition and binding between both types of Duts, explaining why the dimeric but not the trimeric Duts can hydrolyze dUDP as well as dUTP [17]. Therefore, it is surprising that proteins apparently so structurally distant as the dimeric ϕNM1 Dut and the 80α- and ϕ11-trimeric Duts seem to use similar mechanisms for SaPIbov1 de-repression.

Although both families of Duts interact with the SaPI master repressor Stl to activate the SaPI cycle [11], many questions of how the dimeric Duts activate the SaPIbov1 cycle still remain to be deciphered: *i*) do dimeric and trimeric Duts share structural determinants that explain the interaction with Stl?; *ii*) can all the dimeric Duts interact with the SaPI Stl repressor or is this ability confined to a certain subset?; *iii*) does the dUTP also regulate this process? Here, by the structural and functional characterization of different dimeric Dut encoding *S*. *aureus* phages, we provide new insights into these questions.

## Results

### Existence of allelic variants of *S*. *aureus* phage encoded dimeric Dut proteins

**Fig 1:**
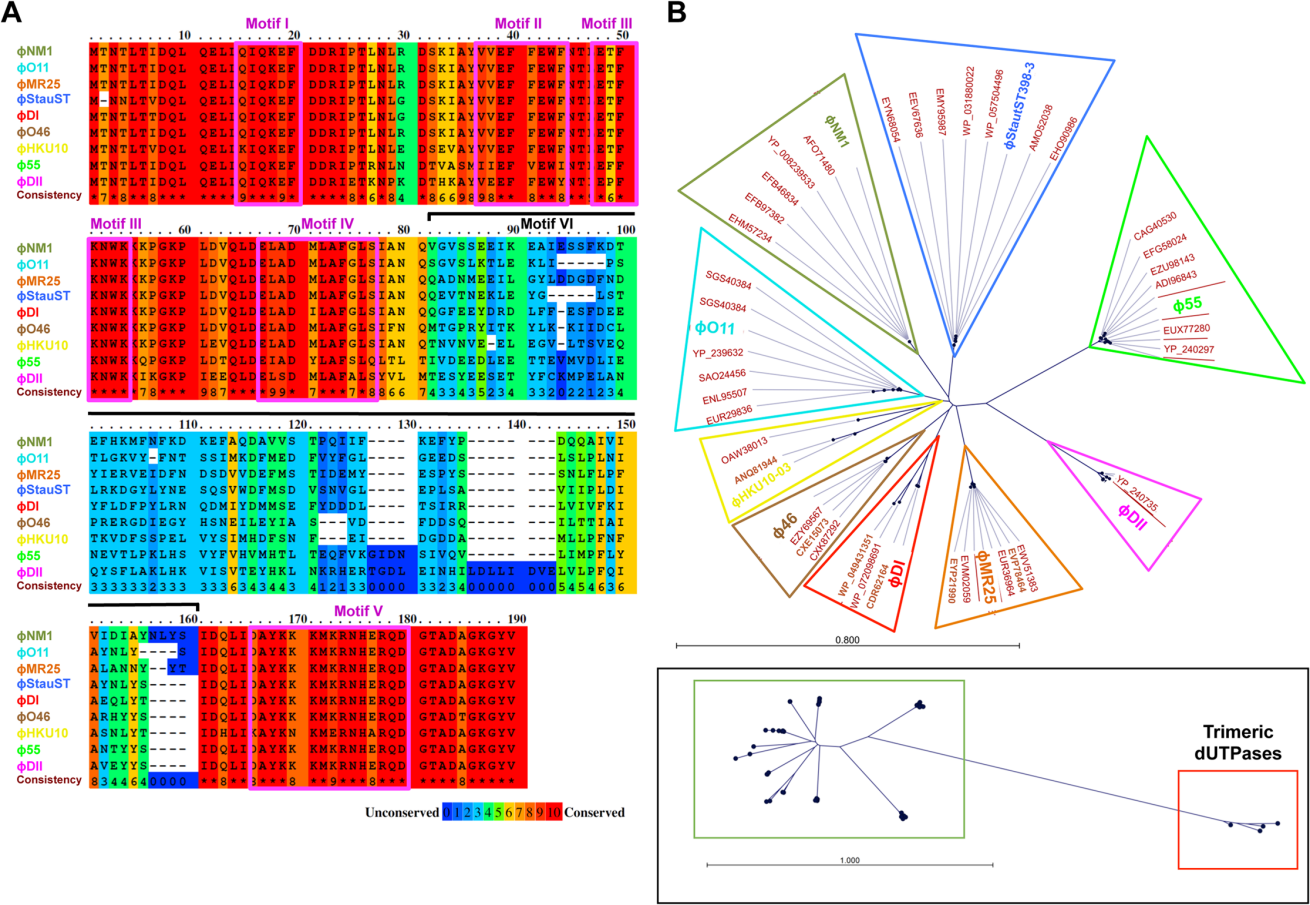
*S*. *aureus* phage coded dimeric Duts show allelic variation. (A) Alignment of dimeric Duts from *S*. *aureus* phages ϕNM1, ϕDI, ϕDII, ϕO11, ϕO46, ϕMR25, ϕStauST398-3 (StauST), ϕHKU10-03 (HKU10) and ϕ55 are shown representing the nine different dimeric Dut families identified in the phylogenetic tree described below (Fig 1B). Colours indicate relative sequence conservation at each position, with red being most conserved and blue being least (alignment generated by PRALINE). The five conserved catalytic motifs in dimeric Duts are highlighted in magenta boxes and labeled. The bracket indicates the localization of the extra motif VI. (B) Radial phylogenetic tree of identified staphylococcal and *Staphylococcus* phage dimeric Duts. Radial tree showing the 9 different groups, constructed using the alignment of protein sequences of the dimeric Duts from staphylococcal phages (including the full sequence, not just the variable region) listed in S1 Table. An NCBI BLAST of the known SaPI inducing ϕNM1 phage encoded dimeric Dut (full sequence) was used to select 59 dimeric Dut sequences that were annotated as staphylococcal phage Duts or as staphylococca*l* Duts. *S*.*aureus* does not encode a genomic Dut so any such annotations would also be phagic. Four trimeric staphylococca*l* phage Duts were also included in the alignment and tree to provide outliers and show the distance of these proteins from the dimeric Duts. The accession numbers for all sequences are listed in S1 Table. The small box below shows the full tree, including the trimeric Dut outliers (red box), which were hidden for clarity in the more detailed tree above. This detailed tree consists of the area highlighted with the green box in the full depiction. Groups are shown by clustered leaf node names and the name of the representative used in the sequence alignment of panel (A) is highlighted with the same colour of the group. The tree was created using the following parameters; algorithm = Neighbour Joining, distance measure = Jukes-Cantor, bootstrap = 100 Replicates. Proteins are named by NCBI accession number with the exception of the group representative where the name of the corresponding phage is included used (S1 Table).

Some *S*. *aureus* phages encode dimeric instead of trimeric Duts. In previous work, we observed that different helper phages encoded allelic variants of the trimeric Duts with differing affinities for the SaPIbov1-encoded repressor [2, 6]. Therefore, we initiated this story by searching for, and analysing, dimeric Duts identified in a subset of 59 staphylococcal phages (S1 Table). These Duts were randomly selected from an NCBI BLAST for the identified ϕNM1 Dut. Some of these Duts were annotated as being from *Staphylococcus* strains, however these are mis-annotated and should be phage Duts as *S*. *aureus* does not encode a dUTPase in its genome [18]. Sequence alignment of the full Dut protein sequences showed the presence of allelic variants in the dimeric Duts (Fig 1A). We exploited these sequence differences to classify the phage encoded dimeric Duts using a distance-based phylogenetic tree. By aligning the full sequence of each of the 59 identified staphylococcal phage encoded dimeric Duts using the neighbor joining method, the dimeric Duts were organized into 9 different families/groups (Fig 1B and S1 Table). Remarkably, sequence alignment of a representative of each of these 9 groups indicated that the allelic variability is accounted for by the presence of a variable central region of about 50 to 60 residues, which is flanked by two regions of high sequence conservation (Fig 1A). The conserved regions encompass the five proposed catalytic motifs present in all the dimeric Duts (Fig 1A and S1A Fig) [8]. By contrast, the central region is variable in sequence and in size (Fig 1A). This follows a parallelism with the sequence organization observed in the allelic variants of the trimeric Duts, where a highly variable central region is also flanked by conserved catalytic motifs [3]. Thus, to maintain consistency with the trimeric Dut motifs, we have named this region as motif VI. It is notable that despite the sequence and predicted structural differences, the dimeric and trimeric staphylococcal phage Duts present similarities, beyond their enzymatic activity, in sequence organization. Both show highly conserved regions with five catalytic motifs, which are completely different for each oligomeric family (S2 Table), flanking a region with high variability in sequence and size.

### Allelic variants of staphylococcal dimeric Duts show different inducing activities

**Fig 2:**
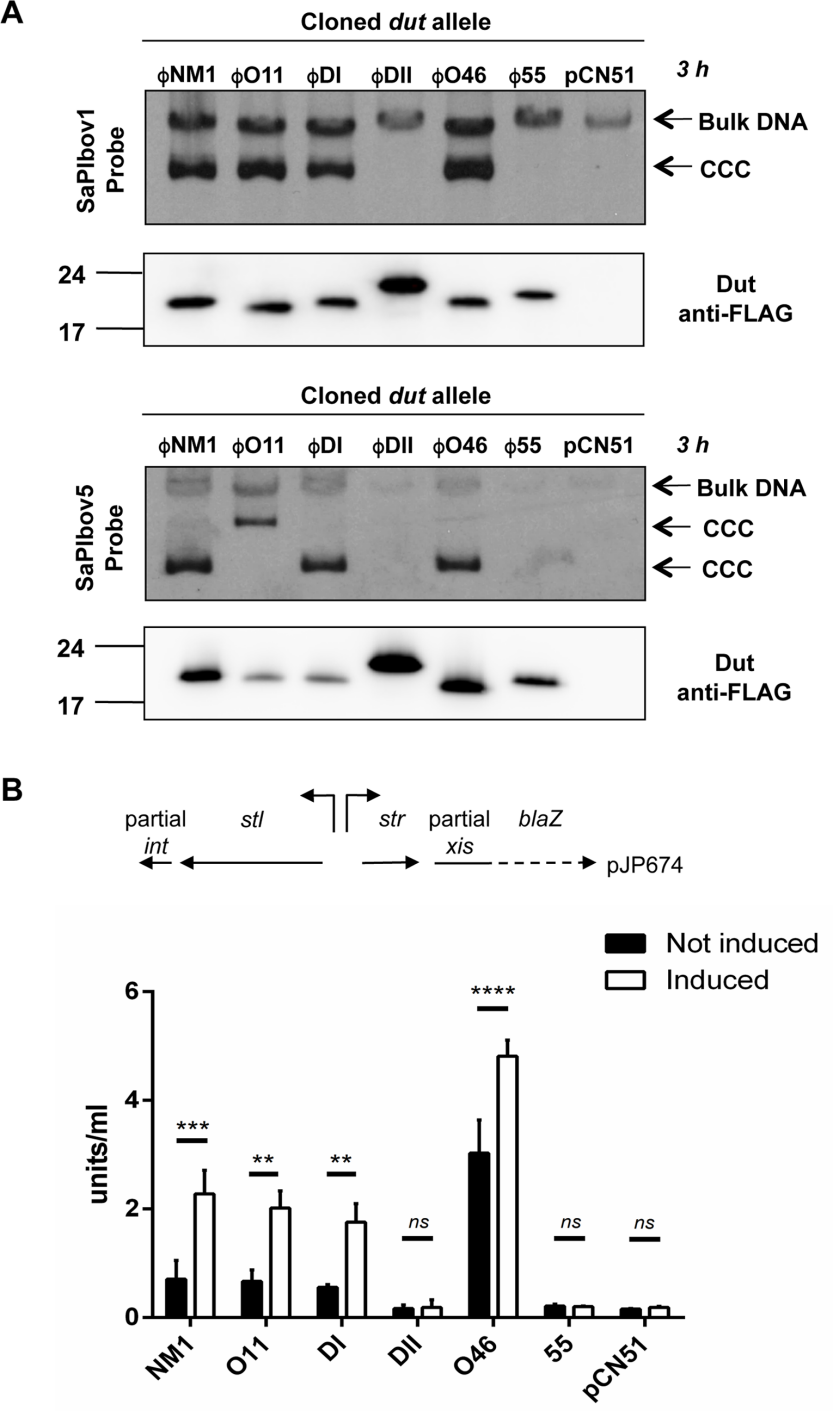
SaPIbov1 replication is induced by different dimeric Duts variants. (A) SaPIbov1 and SaPIbov5 excision and replication following induction of cloned ϕNM1, ϕO11, ϕDI, ϕDII, ϕO46, and ϕ55 *dut* genes. Strain JP6774 containing SaPIbov1 and strain JP16140 containing SaPIbov5 were complemented with the different plasmids expressing the 3xFLAG-tagged dimeric Duts or the empty pCN51 plasmid as a control. Samples were isolated at 3 hours after induction with 3μM CdCl_2_ and Southern blots were performed using a probe for the SaPIbov1 and SaPIbov5 integrase (S4 Table). The upper band is ‘bulk’ DNA, including chromosomal, phage, and replicating SaPI. CCC indicates covalently closed circular SaPI DNA. In these experiments, as no helper phage was present, the excised and replicating SaPI DNA appears as part of the bulk DNA or as CCC molecules, rather than the linear monomers that are seen following helper phage-mediated induction and packaging. The lower panels below each Southern are western blots probed with antibody to the FLAG-tag carried by the Dut proteins. (B) The diagram represents a schematic of a *blaZ* transcriptional fusion generated in pJP674. β-lactamase assays were performed on strains containing pJP674 together with pCN51-derived plasmids expressing the ϕNM1, ϕO11, ϕDI, ϕDII, ϕO46, and ϕ55 *dut* genes or the empty pCN51 control (JP15105). Samples were taken after 5 hours in the absence or following induction with 5μM Cadmium. All data is the result of three independent experiments. Error bars represent SEM. A 2-way ANOVA with Sidak's multiple comparisons test was performed to compare mean differences within rows. Adjusted *p* values were as follows; ϕNM1 = 0.0003***, ϕO11 = 0.0021**, ϕDI = 0.0075**, ϕDII = > 0.9999^ns^, ϕO46 = < 0.0001****, ϕ55 = > 0.9999 ^ns^, pCN51 = > 0.9999 ^ns^.

An interesting feature of the phage trimeric Duts is that the different allelic variants have different affinities for the SaPIbov1 Stl repressor [2, 4, 6], this being a mechanism used by the phages to avoid SaPI induction [5]. We wondered whether the trimeric and dimeric parallelism shown at sequence organization level also extends to their inducing capacities. To test this, we checked the induction capacity of a subset of dimeric Duts, including the Dut encoded by phage ϕNM1, which has previously been shown to induce SaPIbov1 mobilization [11]. The genes expressing 3xFlag-tagged Dut proteins from phages ϕDI, ϕNM1, ϕ55, ϕDII, ϕO46 and ϕO11, representing six different Dut families from the nine identified (Fig 1B), were cloned into plasmid pCN51 under the control of the P*cad* promoter. Note that these Duts were chosen as representatives of the most prevalent families of the *S*. *aureus* phage dimeric Duts. These plasmids were introduced into a SaPIbov1- or SaPIbov5-positive strain, and the induction of the SaPI cycle by the cloned genes was tested. Both SaPIbov1 and SaPIbov5 encode an identical Stl repressor. The Duts from phages ϕDI, ϕNM1, ϕO46 and ϕO11 induced both SaPIbov1 and SaPIbov5, while the Duts from phages ϕ55 and ϕDII induced neither (Fig 2A). As the Dut protein levels produced from these constructs showed equivalent or greater expression of the non-inducing Duts (Fig 2A), we concluded that some dimeric Duts are capable of de-repressing SaPIbov1 and SaPIbov5, while other allelic variants do not display this ability.

To clearly confirm the inducing capacity of this subset of dimeric Duts, we selected ϕDI and ϕNM1 as representative members of inducing Duts and constructed inframe deletions of the phage *dut* gene in their corresponding prophage genomes. These phages were chosen because they use two completely different strategies for packaging. While ϕDI uses the *cos* packaging mechanism, ϕNM1 uses the headful packaging (*pac*) strategy [19]. We next tested the ability of these mutants to induce the SaPIbov5 or SaPIbov1 cycle. As with the phages, we selected these two islands because they encode an identical Stl repressor but use different packaging strategies: SaPIbov5 is a *cos* island, while SaPIbov1 is *pac* [20]. SaPIbov5 was introduced in the lysogenic strains carrying the ϕDI or the ϕDI Δ*dut* mutant, while SaPIbov1 was introduced in the lysogenic strains carrying the ϕNM1 or the ϕNM1 Δ*dut* mutant. The strains were SOS induced (using Mitomycin C) and the SaPI cycle analyzed, showing that the wild-type phages ϕDI and ϕNM1, but not their respective *dut* mutants, induced SaPIbov5 and SaPIbov1, respectively (S2 Fig). Finally, and since both SaPIbov1 and SaPIbov5 carry a *tet*M marker, which facilitate the transfer studies, the lysates generated after SOS induction of the aforementioned strains were analyzed for the presence of infectious phage and SaPI particles. As shown in Table 1, while the wt phages highly transferred the SaPIs, deletion of the Δ*dut* gene significantly reduced SaPI transfer. Moreover, the results showed that the SaPIs, once induced, severely interfered with phage reproduction, clearly confirming that the dimeric Duts are the *bona fide* SaPIbov1 and SaPIbov5 inducers.

**Table 1:** Transfer of SaPIbov1 and SaPIbov5 by dimeric Duts ФD1 and ФNM1.

	No island	SaPIbov1			SaPIbov5		
Phage	Фtitre^a^	Rpl^b^	SaPI titre^c^	titre^a^	Rpl^b^	SaPI titre^c^	Фtitre^a^
ФD1	7.91x10^4^				*+*	3.65 x 10^5^	4.63x10^2^
ФD1Δ*dut*	1.06x10^5^				*-*	<10	3.14x10^5^
ФNM1	3.62x10^9^	*+*	4.47x10^7^	3.56x10^7^			
ФNM1Δ*dut*	4.20x10^9^	*-*	<10	1.35x10^9^			

The means of the results of three independent experiments are presented. Variation was within 5% in all cases.

^a^Plaques ml^-1^ of lysate, using RN4220 as indicator.

^b^Rpl, replication as determined by Southern blot.

^c^Transductants ml^-1^of lysate, using RN4220 as recipient.

### Allelic variants of dimeric Duts directly interact with Stl and induce SaPIbov1 mobilization

**Fig 3:**
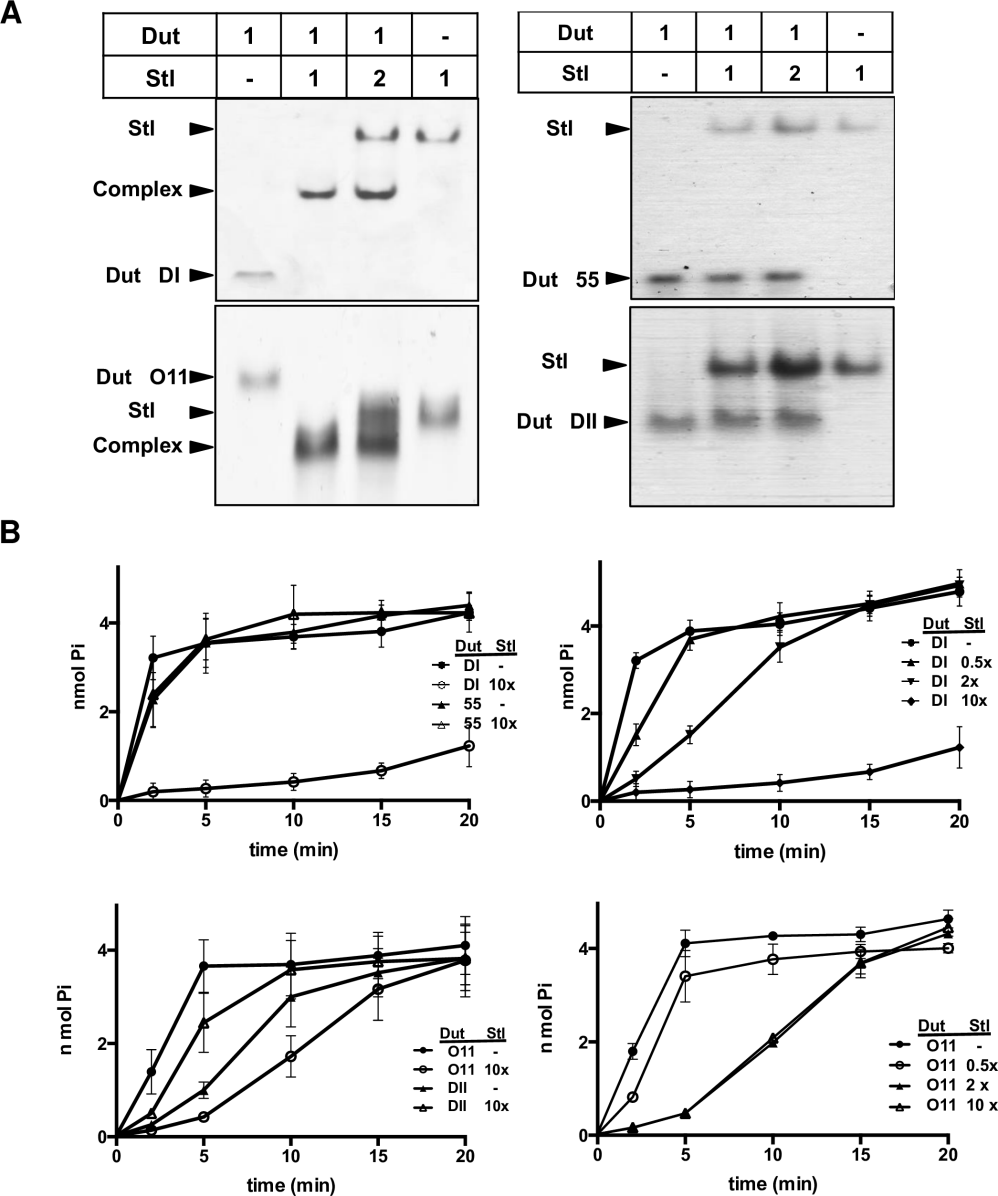
Interaction between Stl and the allelic variants of dimeric Duts from *S*. *aureus* phages and the effect on dUTPase activity. (A) The capacity of the ϕDI, ϕO11, ϕPhi55 and ϕDII dimeric Duts to form a complex with Stl was checked by Native-PAGE maintaining a constant amount of Dut proteins (17 μM) and identical or double concentrations of Stl. (B) Binding of Stl inhibits dUTPase activity. The enzymatic activity of the inducer dimeric Duts ϕDI and ϕO11 were inhibited by Stl whereas it was not affected for the non-inducer allelic variants ϕ55 and ϕDII even though a 10-fold excess of Stl was used (left panels). The dUTPase activity is inhibited by Stl in a dosage dependent manner for ϕDI and ϕO11 Duts (right panels). Average and standard deviation of six replicates are shown.

Trimeric Duts induce the SaPIbov1 cycle through interaction with the SaPIbov1-encoded Stl repressor [2], disrupting the binding of Stl to its target site and inducing transcription of the Stl-repressed SaPIbov1 genes. A similar mechanism has been postulated for the dimeric ϕNM1 Dut [11]. To analyze whether this mechanism is general for all the dimeric Duts with SaPI mobilization capacity, we used the plasmid pJP674, which carries a β-lactamase reporter gene fused to *xis*, downstream of the encoded Stl-repressed *str* promoter and Stl (see Fig 2B). This plasmid was introduced into strains expressing the different cloned Dut proteins, and expression was tested in the presence of an inducing concentration of CdCl_2_. In agreement with the previous results analyzing SaPI replication (Fig 2A), induction of the plasmids expressing ϕDI, ϕNM1, ϕO46 and ϕO11 Dut dimeric proteins increased β-lactamase expression (Fig 2B). In contrast, no variation in β-lactamase expression was observed when the ϕ55 and ϕDII dimeric Duts were expressed. This result suggests that the different allelic variants of dimeric Duts capable of SaPI mobilization induce the SaPI cycle by interacting with the Stl repressor and releasing transcription of Stl-repressed genes, as described for the unrelated trimeric Dut proteins [2, 3, 7]. To further confirm this mechanism we analyzed the direct interaction between Stl and the dimeric Duts by Native-PAGE (Fig 3A). Mixtures of the Stl with ϕDI or ϕO11 Duts, both of which mobilize SaPIbov1, clearly show the generation of new bands corresponding to a Stl-Dut complex, concomitant with the disappearance of the bands corresponding to the individual proteins (Fig 3A), further supporting the hypothesis of a direct interaction of these Duts with the Stl. Titration assays confirmed that the interaction of ϕDI or ϕO11 Duts with Stl is accomplished in a 1:1 molar ratio (S3 Fig). Similar results were previously described for the interaction of the ϕNM1 Dut with Stl [11]. As was expected for the dimeric Duts that cannot induce SaPI mobilization, no bands corresponding to this complex were observed when this assay was carried out with ϕ55 or ϕDII (Fig 3A), confirming that these Duts are incapable of interacting with Stl.

### dUTPase activity and Stl binding

We next analyzed whether the capacity of the different dimeric Duts to de-repress the SaPIbov1 cycle is related to their intrinsic enzymatic activity. To test this we measured the dUTPase activity of the allelic variants of dimeric Dut. As is shown in Table 2, no significant differences were observed in the specific activity and K_M_ values between Duts with or without SaPI inducing capacity. In addition, these catalytic parameters were within ranges observed for dimeric Duts from other organisms [9].

**Table 2:** Nucleotidase activity.

Dut^a^	K_M_ (μM) dUTP	Nucleotidase Specific Activity^b^						SaPI induction ^d^
		dUTP	dCTP	dATP	dGTP	dTTP	dITP	
**ϕDI**	7,4	27	1.03	0.17	0.02	0.49	0.15	+
**ϕDII**	11,1	35	8.74	0.07	NA	0.52	2.03	-
**ϕ55**	13,6	30	1.01	0.04	NA	0.47	0.03	-
**ϕO11**	17,4	22	2.20	NA	NA	0.54	0.05	+
**ϕNM1**	17,5	29	5.84	0.04	NA	0.55	0.16	+
**ϕDI**^**A73L**^	NA^c^	NA	NA	NA	NA	NA	NA	-

^a^His(6)-Dut protein purified.

^b^Specific activity (μmoles/min x mg) measured as production of PPi at 25°C. Variation was within ±10% in all cases.

^c^NA: no activity detected in the experimental conditions used.

^d^(+): inducing SaPI activity; (-): no inducing SaPI activity.

It has been shown that other members of the all-α NTP pyrophosphohydrolases superfamily, to which the dimeric dUTPases belong, have the capability to hydrolyze alternative deoxynucleotides [9]. To further characterize the *S*. *aureus* phage-encoded dimeric Duts, we measured their nucleotidase activity against several dNTPs. We observed that all the tested dimeric Duts have dCTPase activity. This activity does not relate with the SaPI induction capacity of the dimeric Duts since one inducing Dut (ϕNM1) and one non-inducing Dut (ϕDII) present with the higher dCTP hydrolytic activities (Table 2). In addition, the non-inducing ϕDII also presented a considerable dITP hydrolytic activity (Table 2). With these results we conclude that there is no correlation between de-repression capacity and NTPase activity. Furthermore, this analysis reveals that some dimeric Duts from *S*. *aureus* phages have the capacity to hydrolyze alternative dNTP, mainly dCTP, an ability present in other members of the all-α NTP pyrophosphohydrolases superfamily, but absent in the previously characterized dimeric Duts [9, 21, 22].

Binding of Stl to trimeric Duts inhibits dUTPase activity [6, 7]. Furthermore, this interaction was responsible for the Stl-induced dUTPase activity inhibition reported for the dimeric ϕNM1 Dut [11]. Therefore, we analyzed if this Stl interaction-induced activity inhibition also occurs with other dimeric inducing dUTPases. For this purpose, we chose two of the allelic variants that showed Stl binding and SaPIbov1 mobilization capacity (ϕDI and ϕO11) and two lacking these capacities (ϕ55 and ϕDII). Incubation of both Duts with an excess of Stl (10x) highly inhibited or completely abolished the dUTPase activity of the ϕO11 and ϕDI Duts, respectively (Fig 3B). Conversely, no effect on the hydrolytic activities of the non-inducing ϕ55 and ϕDII Duts was observed (Fig 3B). Titration assays with variable concentrations of Stl further confirmed that ϕDI and ϕO11 dUTPase activity inhibition is proportional to Dut-Stl interaction (Fig 3B).

### Structural characterization of an inducing phage dimeric Dut

**Fig 4:**
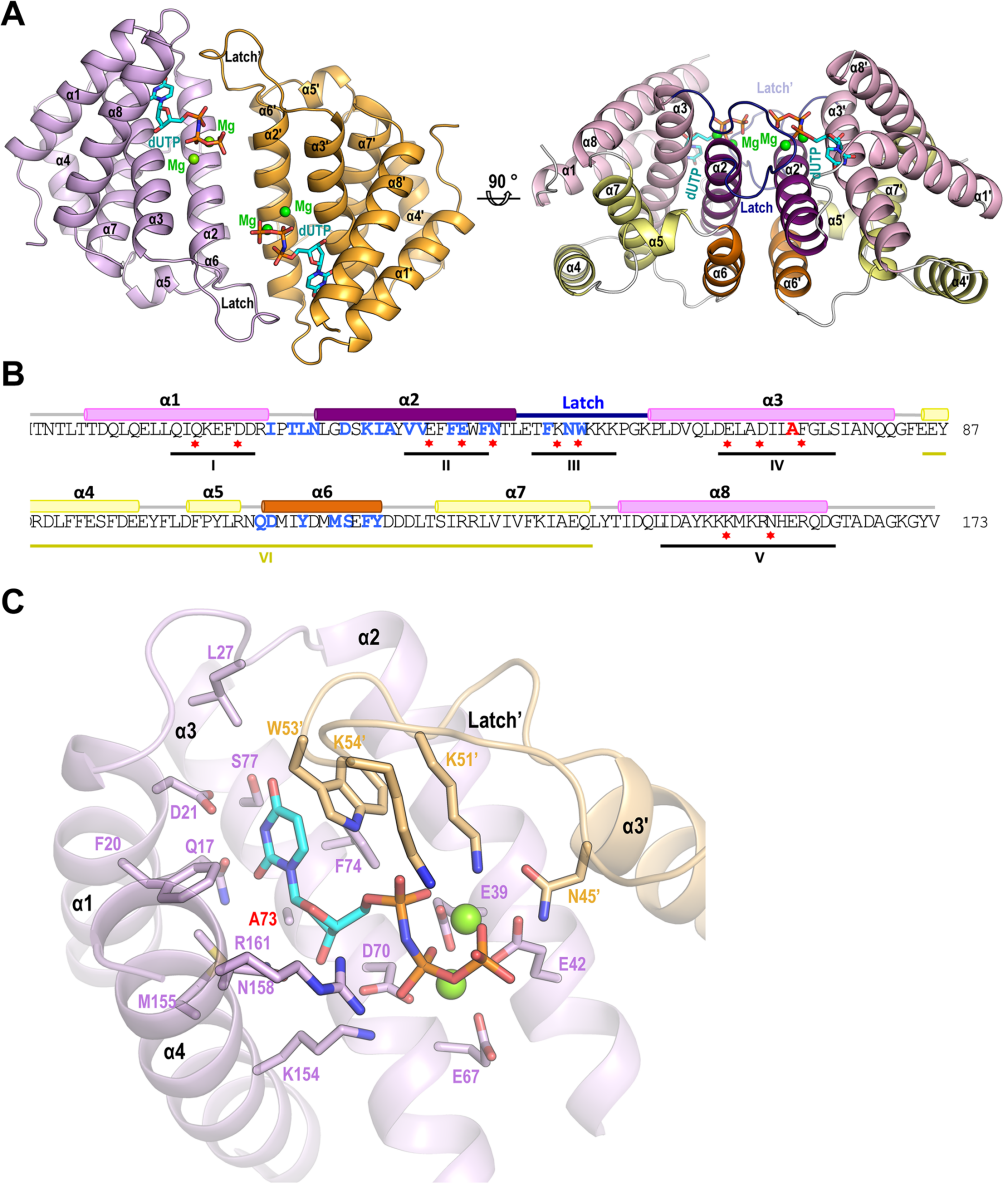
Structure of ϕDI dimeric Dut. (A) Cartoon representation of the ϕDI Dut dimer with protomers coloured in pink and orange, respectively (left). The secondary structural elements are numbered and labeled in order from the N to C terminus (the ‘ indicates elements from the second protomer). A molecule of dUPNPP (sticks coloured by atom type) and two Mg ions (green spheres) occupies the active center of each protomer. In an orthogonal view (right) the conserved and variable (motif VI) regions of the dimeric Duts from *S*. *aureus* phages are colored in pink and yellow tones, respectively. The structural elements exploited to dimerize (helices α2 and α6, and the latch) are highlighted in dark tones. Notice that the nucleotide binding sites and the motifs VI map in opposite faces of the dimer. (B) Sequence of the ϕDI Dut, with residues that interact with the substrate highlighted with red stars and residues that interact across the dimer interface in blue text. The Ala residue mutated in the ϕDI^A73L^ is indicated in red. The locations of the five conserved motifs in the dimeric Duts are indicated. Structural elements are shown above the sequence coloured as (A) right panel. (C) Detailed view of the ϕDI Dut active center. The substrate dUPNPP is represented in stick with carbon atoms in cyan. The residues interacting with the nucleotide are shown in stick representation, with carbon atoms coloured according to the protomer to which they correspond and are labeled with a similar color text with the exception of A73 that is highlighted with red text. Nitrogen, oxygen, phosphorus atoms are coloured in dark blue, red and orange, respectively. The Mg ions are represented as green spheres.

Since none of the *S*. *aureus* phage encoded dimeric Dut structures have been solved yet, and to gain insight into the molecular mechanism of dimeric Dut-Stl interaction and the possible effect of the nucleotide in this process, we accomplished the structural characterization of the inducing dimeric Dut from phage ϕDI in the presence of the nucleotide (Table 3). The structure of the ϕDI Dut in complex with dUPNPP (non-hydrolysable analog of dUTP) and Mg^2+^ was solved by SAD to 2.1 Å resolution (Table 3). The structure showed 4 molecules in the asymmetric unit organized as two independent homodimers with identical conformation (RMSD of 0.32 Å for the superimposition of the dimers) (Fig 4A, Table 3). Similarly, each protomer within the independent homodimers was virtually identical (RMSD < 0.18 Å), with the active center occupied by one dUPNPP molecule and two Mg^2+^ atoms (Fig 4A). ϕDI Dut is an all-helix protein, as previously reported for the dimeric Duts (S1B Fig) [8, 14–16], but uniquely it represents a reduced version of this type of Dut, since each protomer is made up of only eight α-helices (α1, residues 7–23; α2, 29–47; α3 61–82; α4, 86–98; α5, 104–108; α6, 110–121; α7, 127–141 y α8, 144–163), in contrast to the 10 to 13 α-helices and, in some cases additional β-strands, observed in the known dimeric Dut structures (Fig 4A and 4B and S1A Fig). These differences are mainly localized to the C-terminal part, where the ϕDI Dut is shorter by more than fifty residues (S1A Fig), and also at the here-defined motif VI, a region that is not only variable in size and sequence among *S*. *aureus* phage Duts but also among all the dimeric Duts (Fig 1A and S1 Fig). This region, which is well defined in the ϕDI structure, encompasses four helices (α4-α7) and occupies the back part of the nucleotide-binding site (Fig 4A).

**Table 3:** Crystallographic statistics of Dut ϕDI structures.

	dUPNPP	SeMet dUPNPP	*Apo*	A73L Mutant
**Data collection**				
Beamline	DLS-I.04	ALBA-XALOC	DLS-I.04	ALBA-XALOC
Wavelength (Å)	0.97942	0.97925	1.0721	0.97948
Space group	P2_1_	P2_1_	P 2_1_2_1_ 2	P6_1_ 2 2
Cell dimensions (Å)	a = 69.89	a = 70.45	a = 148.42	a = b = 79.45
	b = 81.44	b = 81.23	b = 51.94	c = 132.81
	c = 81.53	c = 82.54	c = 43.64	α = β = 90
	α = γ = 90	α = γ = 90	α = β = γ = 90	γ = 120
	β = 106.92	β = 107.41		
Resolution (Å)^a^	81.44–2.10	81.23–3.00	49.03–1.85	68.81–1.90
	(2.21–2.10)	(3.16–3.00)	(1.95–1.85)	(2.00–1.90)
Unique reflections	50736 (7325)	17945 (2608)	29551 (4063)	19182 (2723)
Completeness (%)	99.24 (98.9)	99.9 (99.9)	99.5 (96.5)	99.9 (100)
Multiplicity	4.0 (3.7)	7.0 (7.2)	11.0 (5.2)	9.3 (9.4)
I/σ(I)	8.4 (2.1)	10.8 (2.2)	18.7 (2.2)	11.1 (2.3)
R _merge_	0.048 (0.35)	0.064 (0.45)	0.024 (0.35)	0.040 (0.39)
**Refinement**				
R_work_	0.224		0.176	0.165
R_free_	0.265		0.219	0.209
Number of atoms				
Protein	5388		2631	1381
Ligand	112		-	-
Water	210		181	103
Others	8		-	25
Rmsd, bond (Å)	0.016		0.017	0.007
Rmsd, angles (°)	1.34		1.52	0.924
Ramachandran plot				
Preferred (%)	99		99	98.2
Allowed (%)	1		1	1.8
PDB accession code	5MYD		5MYF	5MYI

^a^Numbers in parentheses indicate values for the highest-resolution cell

R_merge_ = Σ (I -<I>) / Σ<I>

R_factor_ = Σ‖Fo|−|Fc‖/Σ|Fo|

R_free_ is the R_factor_ calculated with 5% of the total unique reflections chosen randomly and omitted from refinement

Based on the conformational movements observed in the dimeric Duts, the protomers have been divided into a rigid and a flexible domain. The rigid domain is involved in dimerization and the flexible mobile domain, the disposition of which is modulated by the substrate, undergoes conformational changes from the “open” ligand-free conformation to the ligand-bound or “closed” conformation [14, 15]. In ϕDI, the mobile domain is reduced to the first (α1) and the last (α8) α–helices due to the shortened C-terminal region, and adopts a “closed” conformation approaching the dUPNPP (S4 Fig). ϕDI dimerization is mediated by the reciprocal helix–helix interaction of helices α2 and α6 from each protomer in the rigid domain (S5 Fig), and is further stabilized at the tips of this interface by the loops connecting helices α1-α2 and α2-α3 (Fig 4A and 4B). The α2-α3 loop that has been termed the “latch” contains catalytic residues and projects onto the active site of the neighboring protomer (see next section) (Fig 4). Altogether, the protomer-protomer interface buries 1246 Å^2^ of the surface and involves 32 residues from each protomer.

### Active site architecture

The ϕDI dimer has two active centers with each one occupied by a molecule of dUPNPP and two Mg^2+^ ions. Both active centers are placed at the same face of the dimer and are connected, forming a long groove delimited by helices α1, α2, α3, and α8, the latch, and the loop α1-α2 (Fig 4A). Five catalytic motifs conforming the active center are observed in the previous dimeric Dut structures [8, 14, 16], of which only four (motif I-IV) are strictly conserved in ϕDI (S1A Fig). Motif V is reduced to a single helix (α8) in the *S*. *aureus* phage dimeric Duts (Fig 4B and S1A Fig). Helix α8 conserves some of the characteristic dUTP-interacting positions of motif V, mainly the Asn (Asn158) that anchors the deoxyribose and the two interactions with phosphates mediated by Lys154 and Arg161 (Fig 4B and 4C). However, the C-terminal truncation eliminates several additional contacts with the dUTP phosphates observed in other dimeric Duts. These contacts are partly recovered by novel interactions specific to the *S*. *aureus* phagic Duts, provided by Asn45 or Lys59 from the neighbouring protomer, which are not included in any of the previously defined conserved motifs (Figs 4B and 4C and S1A). These novel crossed interactions are allowed by the rearrangement of the protomer-protomer disposition, with helices α2 and α6 in a more parallel orientation than in other dimeric Dut structures (S5 Fig). This protomer-protomer reorientation reduces the dimerization interface (~1240 Å^2^) with respect to the interface observed in other dimeric Dut structures (~1900–1600 Å^2^).

Despite these differences that could indicate some catalytic peculiarities, dUTP-interacting residues from motifs I-IV and partly from motif V present a highly conserved spatial disposition, supporting that phagic dimeric Duts follow a mechanism of reaction similar to that proposed for the dimeric Dut family [15]. Additionally, one more peculiarity is found at the sugar moiety binding site. In the previously characterized dimeric Duts, the deoxyribose was sandwiched between the two aromatic rings of a conserved His-Phe couple in motif IV. This couple precludes ribose access to the sugar binding-site since the extra hydroxyl group would produce clashes with the His and Phe rings. Furthermore, the His is the residue proposed as responsible for specificity towards uracil [8]. Surprisingly, in phagic dimeric Duts, the His is substituted by an Ala (Ala73 in ϕDI) (Fig 4B and 4C and S1A Fig). This substitution expands the sugar binding pocket, lowering the steric restrictions for accommodating other nucleotides as substrate, explaining the observed dCTPase and dITPase activity showed by some allelic forms.

### The dUTP nucleotide, but not the dUMP, inhibits the Dut-Stl interaction

**Fig 5:**
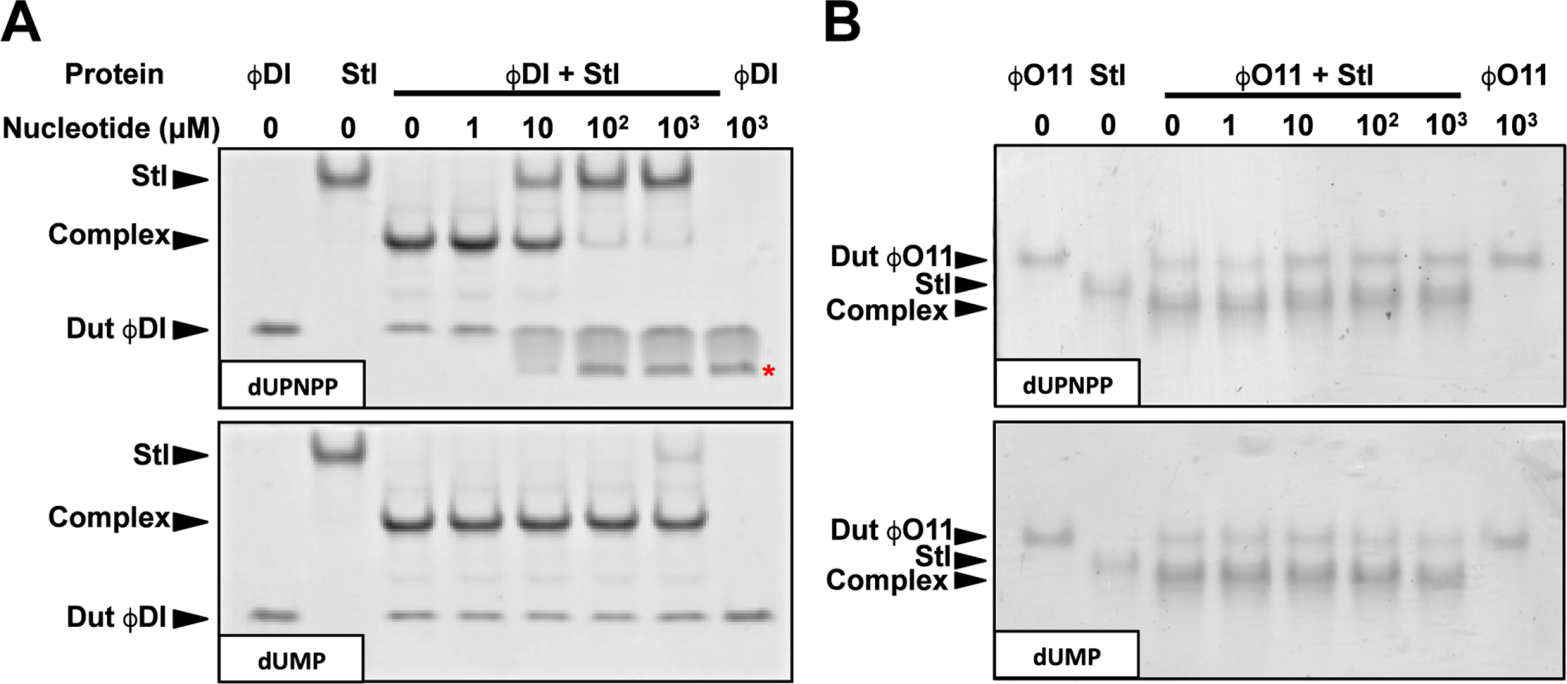
The dUTP substrate but not the dUMP product reduces the Dut-Stl interaction. Native-Page gels were performed to analyze the effect of the substrate and product on the interaction of two inducing dimeric Duts, (A) ϕDI and (B) ϕO11, with Stl. Increasing concentrations (from 0 to 1000 μM) of dUPNPP (top gels) or dUMP (down gels) were added to equimolecular concentrations of each Dut with Stl, and the Dut-Stl complex formation was evaluated by Native-PAGE. Notice the reduction of the Stl-Dut complex band when the dUTP analogue but not the dUMP product was present. For ϕDI a new band (labeled with a red asterisk) corresponding to the complex between dUPNPP and the Dut can be observed when the Stl-Dut complex disappears. For each experiment a representative gel of 3 independents assays is shown.

Others and ourselves have demonstrated that dUTP inhibits the interaction of the SaPIbov1 Stl with *S*. *aureus* trimeric Duts [6, 7]. Binding to the dUTP induces a conformational change in trimeric Duts dependent on the conserved C-terminal P-loop (motif V of the trimeric Duts), which covers the active site once the nucleotide is accommodated [3, 23], blocking the access of Stl to its binding site [6, 7]. Since the SaPIbov1 Stl has an impact on the dUTPase activity of inducing Duts, we wondered if this worked in reverse; whether the ligand for the Duts, dUTP, has an effect on the binding of the dimeric Duts to Stl. Thus, we analyzed whether dUTP acts as a regulator for the dimeric Duts, using Native-PAGE with the inducing ϕDI and ϕO11 Duts and dUPNPP, the non-hydrolysable analog of dUTP, as substrate. Remarkably, dUPNPP proved capable of competing away the dimeric ϕDI Dut from binding to the SaPI Stl repressor, with an IC_50_ of 13.9 μM (Fig 5A and S6 Fig). In contrast, only a weak reduction in ϕDI Dut-Stl binding was observed when high concentrations (1 mM) of the product, dUMP, was present (Fig 5A). Similarly, the ϕO11 Dut-Stl complex formation is also inhibited by dUPNPP, but to a lesser extent than for ϕDI (Fig 5B), in clear correlation with the lower binding capacity of Stl to this dimeric Dut as indicated by the dUTPase inhibition tests (Fig 3B). As is the case for ϕDI, the product dUMP has not effect on ϕO11 Dut-Stl binding (Fig 5B). These observations represent another striking similarity between the dimeric and trimeric staphylococcal Duts, since in both families the interaction with Stl seems to be inhibited by the substrate dUTP but not by the product dUMP.

**Fig 6:**
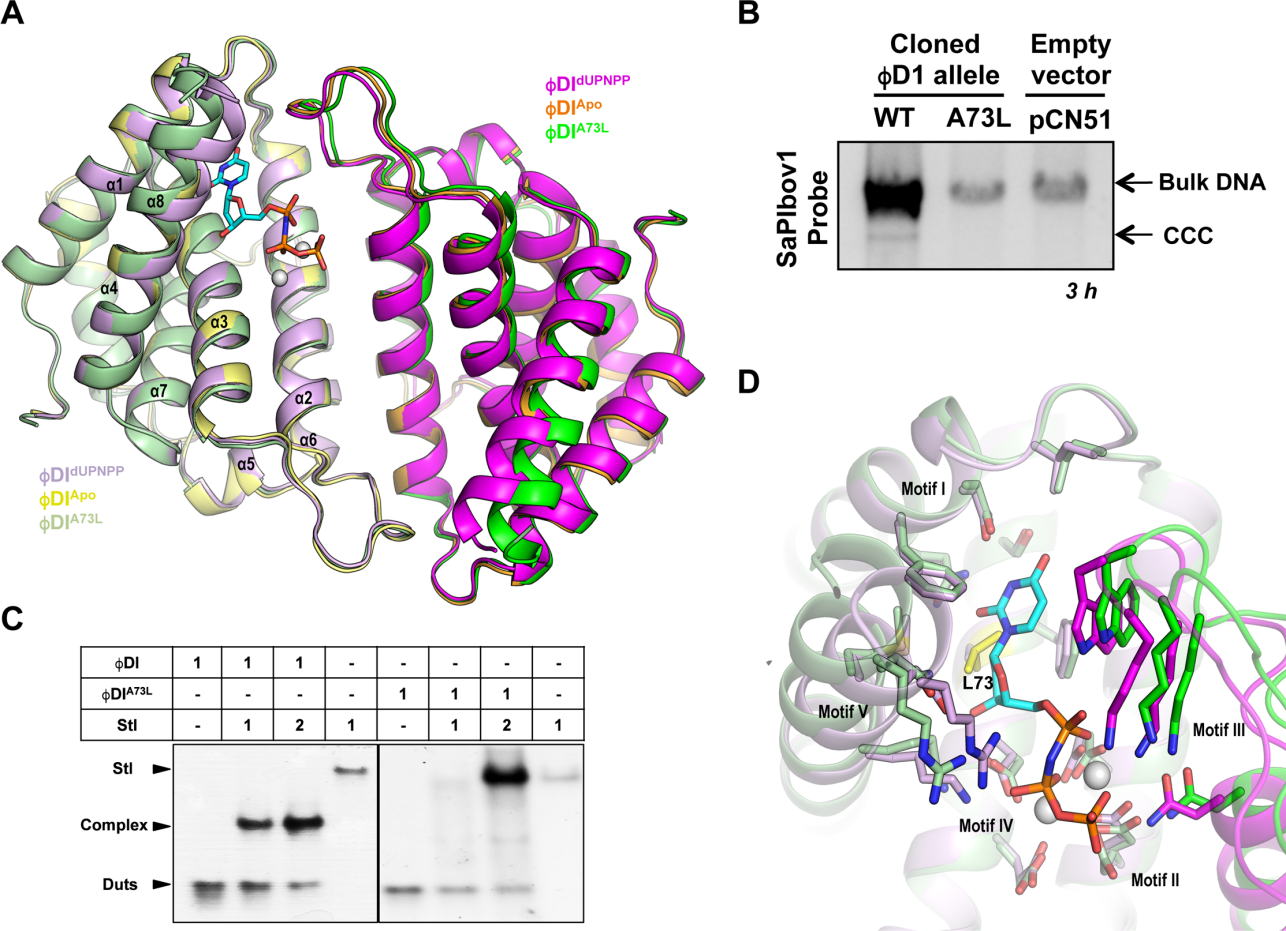
Structures of ϕDI *apo* and ϕDI^A73L^ dimeric Duts showed minimal conformational changes in the mutant, but a high impact on SaPI induction and Stl interaction. (A) Superimposition of three-dimensional structures of the ϕDI dimer in *apo* form (yellow tones), dUPNPP bound (magenta tones) and as A73L mutant (green tones) in cartoon representation. The nucleotide in the ϕDI-dUPNPP structure is represented in stick with carbon atoms in cyan and Mg as green spheres. (B) SaPIbov1 excision and replication following induction of the cloned ϕDI^A73L^ *dut* gene. Strain JP6774 containing SaPIbov1 was complemented with plasmids expressing the 3xFLAG-tagged ϕDI and ϕDI^A73L^ dimeric Dut or the empty pCN51 plasmid as a control. Samples were isolated at 3 hours after induction with 3μM CdCl_2_ and Southern blots were performed using a probe for the SaPIbov1 integrase (S4 Table). The upper band is ‘bulk’ DNA, including chromosomal, phage, and replicating SaPI. CCC indicates covalently closed circular SaPI DNA. In these experiments, as no helper phage was present, the excised and replicating SaPI DNA appears as part of the bulk DNA or as CCC molecules, rather than the linear monomers that are seen following helper phage-mediated induction and packaging. (C) Native-PAGE experiment was performed to analyze the binding capacity of the ϕDI^A73L^ Dut vs the ϕDI wild-type form. Proteins were mixed with Stl in equimolecular relationship (in monomers). A clear reduction in the band corresponding to the Stl-ϕDI Dut^A73L^ complex with respect to the Stl-ϕDI Dut complex is observed. (D) Close-up view of the superimposed ϕDI-dUPNPP and ϕDI^A73L^ active centers. Residues responsible for substrate coordination and binding are represented in sticks with carbon atoms colored according to the structure to which they correspond. The Leu73 residue at the bottom of the active center of the ϕDI^A73L^ mutant that precludes the binding of the nucleotide is colored in yellow. The substrate dUPNPP in the ϕDI-dUPNPP structure is represented in stick with carbon atoms in cyan and Mg ions are represented as grey spheres.

Since our data indicates that dUTP also inhibits the binding of Stl to dimeric Duts and following the parallelism between both types of Duts, we hypothesized that dimeric Duts could undergo conformational changes related to inducing capacity. This idea was also supported by the observation that previously characterized dimeric Duts alternate between “open” (ligand-free) and “closed” (ligand-bound) conformations [14, 16] (S4 Fig). To validate this idea, we obtained the three-dimensional structure of ϕDI Dut in *apo* form (Table 3), allowing us to compare this with the dUPNPP bound structure. The *apo* ϕDI structure showed a dimeric organization that, surprisingly, presents a “closed” conformation almost identical to the dUPNPP-bound form (Fig 6A). This was confirmed by the minimal differences observed when both dimers were superimposed (RMSD of 0.36 Å). The major differences are observed at the C-terminal helix α8, where electron density is poorly defined for the *apo* structure’s last fourteen residues, indicating that the region is highly flexible. Similarly, the last five residues of helix α8 of the dUPNPP-bound structure present poor density, confirming the high intrinsic mobility of this region. These minimal changes could represent the transition between “open” and “closed” conformations in phagic dimeric Duts. Although to a lesser extent, the nucleotide seems to stabilize the C-terminal helix of the phagic dimeric Duts, similarly to the dUTP-induced ordering of C-terminal motif V in the trimeric Duts.

Although the structural results showed only minimal dUTP-mediated conformational changes, we then analyzed if these changes had an impact on Stl binding. This idea was based on our previous results with the trimeric Duts, where modifications disturbing the correct folding of motif V interfered with Stl binding and SaPI de-repression [3, 6]. To test this possible parallelism we designed a ϕDI Dut mutant unable to bind the dUTP nucleotide. This mutant would be similar to that generated in the trimeric 80α Dut (Dut Y84I), which, as previously mentioned, was unable to bind the dUTP nucleotide and did not induce SaPI mobilization [3]. Based on the crystallographic data, we decided to mutate Ala73 to the hydrophobic residue Leu. As we previously indicated, Ala73 is placed at the bottom of the nucleotide binding pocket occupying the position responsible for deoxyribose discrimination (Fig 4C), thus its substitution for a bulky hydrophobic residue (Leu) should abolish nucleotide access.

*In vitro* characterization confirmed that the ϕDI A73L mutant (ϕDI^A73L^) presented a null dUTP-binding capacity when it was checked by ITC (S7 Fig) and, consequently, was inactive as dUTPase (Table 2). Remarkably, and in support of our hypothesis, ϕDI^A73L^ did not induce SaPIbov1 and presented a clear reduction in its capacity to interact with Stl, as confirmed *in vitro* by Native-PAGE assay (Fig 6B and 6C). These results could indicate that the A73L mutation induces a Dut conformation with reduced competence for Stl binding or, more drastically, could impair its correct folding. To evaluate these possibilities, we structurally analyzed ϕDI^A73L^. The three-dimensional structure of ϕDI^A73L^ proves that the protein is well folded, showing a dimeric arrangement generated by crystallographic symmetry (Table 3). Superimposition with wild-type ϕDI structures, both *apo* and dUPNPP forms, confirms an identical folding (RMSD < 0.5 Å; Fig 6A) and rules out any conformational impact of the A73L mutation. A close up view of the active center shows that the new Leu is accommodated without steric difficulties in the nucleotide pocket, with low impact on the surrounding catalytic residues (Fig 6D). However, the C-terminal helix α8 presents a higher stabilization compared not only with the *apo* form but also with the nucleotide complex, allowing us to model all the residues except the last four (Fig 6A and 6D). In this way, the A73L mutation seems to mimic the nucleotide stabilizing of motif V in the trimeric Duts. Although, these conformational changes are extremely modest in comparison to those observed for dUTP-induced motif V stabilization in the trimeric Duts, the parallelism observed between both types of Duts is at least striking given their completely different structures. Taken together, these results suggest that dUTP-induced inhibition of Stl interaction could be mediated by the stabilization of the active center and the catalytic elements. Our results also highlight the idea that two structurally unrelated types of Dut interact with the same Stl repressor by an analogous mechanism involving dUTP as the signaling regulator.

## Discussion

We have shown in this paper that the dimeric and trimeric Duts from *S*. *aureus* phages, despite being structurally antagonistic, present a striking parallelism in their mechanism for SaPI mobilization and escape: *i*) both types of Duts combine highly conserved catalytic motifs characteristic of each type of Dut, which define their own structural scaffold [9, 13], with an internal region highly variable in sequence and length that we have named motif VI [2, 12] (Fig 1); *ii*) both induce SaPIbov1 mobilization by interacting with Stl [2, 11] (Figs 2 and 3); *iii*) the substrate dUTP, but not the product dUMP, modulates the interaction between Dut and Stl [6, 7] (Fig 5); *iv*) perturbation of the Dut dUTP binding sites hampers the interaction with Stl [3, 6] (Fig 6), and *v*) the binding of Stl inhibits Dut catalytic activity [6, 7, 11] (Fig 3). These findings could suggest that dimeric and trimeric Duts present a similar mechanism of interaction with the Stl repressor. However, it has been shown that trimeric Duts interact with Stl as a trimer, while it has been proposed that binding to Stl leads to dimer disruption in the dimeric ϕNM1 Dut [11]. This difference suggests some peculiarities in the molecular mechanism of Stl recognition and binding for each type of Dut. In this way, the modest structural impact of the union of dUTP on dimeric Duts *versus* the crucial role played by the substrate in the structural stabilization of the P-loop motif V in trimeric Duts, a key element in Stl binding inhibition, would support these mechanistic differences. The similarities reinforce the role of dUTP as a signaling molecule, representing a new nucleotide with a second messenger function. Altogether, this indicates that both types of Duts show a conceptually similar regulatory mechanism, where the dUTP works as a switch turning off the interaction with the target, although there may be differences in the molecular way of exerting this mechanism. A detailed characterization at a molecular level, including the determination of the three-dimensional structures of Stl in complex with trimeric and dimeric Duts, is required to elucidate this point.

Phylogenetic classification of the dimeric Duts proposed three separated branches, nucleating dimeric Duts from *S*. *aureus* phages on one of these branches [8]. The crystal structure of the ϕDI Dut described here represents the first structural characterization of this group of dimeric Duts, revealing particular features. As anticipated by the presence of the conserved motifs, the ϕDI Dut maintains the structural core of dimeric Duts, formed by four α helices that conform the active centre and mediate enzyme dimerization. However, the ϕDI Dut represents a simplified version of this family of enzymes, mainly due to the shortening of the C-terminal portion. This reduction in size has functional implications since it affects several residues of motif V that mediate contacts with dUTP phosphates. These catalytic residues are provided in the ϕDI Dut by the neighboring protomer in the dimer. The conservation of these residues in dimeric Duts from *S*. *aureus* phages indicates this feature as representative of this group of enzymes. In the previously characterized dimeric Duts, substrate binding induces a closing movement that brings the C-terminal portion of the enzyme to the dimerization region. Oppositely, the binding of the nucleotide seems to have minimal conformational effect in the ϕDI Dut, which presents a “closed” conformation in its *apo* state. The contacts with nucleotide phosphates have been proposed as an important inductor for closure of the enzyme [16]. Therefore, the absence of the major part of the mobile C-terminal portion, which also provides several phosphate interacting residues, would explain the fixed closed conformation observed for the ϕDI Dut. The replacement of these contacts for new catalytic residues coming from the adjacent subunit requires a protomer-protomer reorientation with respect to the orientation observed in other dimeric Duts. This new relative disposition of the protomers reduces the interface of dimerization, which may decrease the stability of the dimer. Since it has been proposed that dimeric Duts interact with the Stl repressor as monomers [11], it is tempting to speculate that Stl has taken advantage of this low dimer stability to form a heterocomplex with the Dut in its monomeric form. Following this speculation, the differences observed in the capacity for interaction with Stl and for induction of SaPI mobilization shown by the phage dimeric Dut allelic variants could be explained by differences in dimer stability. In addition, the dUTP-mediated reduction of Stl binding shown in the ϕDI Dut would be in line with this proposition, since dUTP would be working as a dimer stabilizer, by interacting with both protomers at the same time. In this context, it is challenging to explain how a mutation that precludes dUTP binding (A73L) also decreases the interaction with Stl. However, the structural characterization of the ϕDI Dut A73L mutant has shown that this mutation promotes a compact conformation that could be stabilizing the dimer, supporting our proposition. During the review of this manuscript, Dokland and collaborators published that Stl interaction with the dimeric Dut from phage ϕNM1 was inhibited by dUTP in close agreement with our results and proposition [24]. However, the authors indicate that the product dUMP also has an inhibitory capacity for the Stl-ϕNM1 Dut interaction, in contrast to the results obtained here for two alternative inducing dimeric Duts. Undoubtedly, the evaluation of this discrepancy and the confirmation of our proposals will require the future molecular characterization of the interaction of Stl with different allelic variants of dimeric Duts from *S*. *aureus* phages.

It is an intriguing question as to why *S*. *aureus* phages encode trimeric or dimeric Duts. The fact that all phage encode functional Duts suggests these enzymes are important for the phage cycle, although these enzymes are dispensable in laboratory conditions, as we have demonstrated here and previously [2]. Our hypothesis is that the phage-encoded Duts perform regulatory functions by interacting with other bacterial or phage proteins to provide the phage with functional advantages, as has previously been shown for other phage-encoded SaPI de-repressing proteins such as Sri, which regulates the cellular helicase loader DnaI [25, 26]. SaPIs seem to have exploited this fact, developing Stl repressors that may have merged to the structure of one of these cellular partners, which would represent a fascinating example of molecular mimicry [1]. Molecular mimicry is widely used by pathogens, particularly viruses, to hijack cellular processes [27], thus in this case, the SaPI would be working as a phage pathogen and the Stl would be a mimetic protein with anti-mimetic activity [1, 10]. The interaction of the phage protein with Stl is highly detrimental for the phage, thus the phagic protein should evolve rapidly, presenting high variability. The phagic Duts are a paradigmatic example of this fact, presenting an extremely variable motif VI and, furthermore, being substituted by analogous proteins (dimeric-trimeric). As a result of this arms race between phages and SaPIs, an amazing variety of trimeric and dimeric Duts have been generated, which show different affinities for the Stl repressor [2, 5]. It might be thought that the dimeric Duts would have won this race, as our data shows that a significant number of these Duts have been able to bypass Stl interaction and thus avoid SaPI induction. However, we think this may not be the case, since our hypothesis is that the decrease in affinity for Stl would also reflect a decreased affinity for the true cellular target proteins. This fact would entail a loss of the functional advantages compared to those phages with greater affinity for these targets. Otherwise, all phages would tend to lose the *dut* gene or would have been able to evolve Duts with low or null affinity for StI. In this way, the huge divergence in sequence and type observed among *S*. *aureus* phage Duts reflects the trade-off between the regulatory advantages in the phage cycle and the detrimental SaPI inductive capacity provided by these proteins. If we define virus species as a polythetic class of viruses that constitutes a replicating lineage and occupies a particular ecological niche [28], it would be worth speculating that this trade-off could have a significant role in phage evolution and speciation. The mutation or acquisition of a non-cognate *dut* gene to escape from SaPI induction would alter the function of the proteins targeted by the Dut, which in turn would affect the phage cycle. To persist in nature, this chimeric phage would have to evolve by introducing compensatory mutations in other parts of its genome to adapt to the presence of the new regulator. In this way, a set of phage genes would be diverging and, if this genetic combination is viable, a distinct population may then become a new species. This fascinating hypothesis is currently under study.

## Methods

### Bacterial strains and growth conditions

S3 Table lists the bacterial strains used for this study. The procedures for preparation and analysis of phage lysates, in addition to transduction and transformation of *S*. *aureus*, were performed essentially as previously described [29, 30]. *S*. *aureus* was grown in Tryptic soy broth (TSB) or on Tryptic soy agar plates. *E*. *coli* was grown in LB broth or on LB agar plates. Antibiotic selection was used where appropriate.

### DNA methods

General DNA manipulations were performed using standard procedures. The oligonucleotides used in this study are listed in S4 Table. The labeling of the probes and DNA hybridization were performed per the protocol supplied with the PCR-DIG DNA-labelling and Chemiluminescent Detection Kit (Roche). Detection probes for SaPI DNA in Southern blots were generated by PCR using primers SaPIbov1-112mE and SaPIbov1-113cB (SaPIbov1 and SaPIbov5) as listed in S4 Table.

### Plasmid construction

S5 Table lists the plasmids used for this study. The plasmid constructs expressing the different Dut proteins were prepared by cloning PCR products obtained using the oligonucleotide primers listed in S4 Table. All clones were sequenced by the IBV Core Sequencing facility or Eurofins genomics. Dut proteins were expressed in *S*. *aureus* under inducing conditions from the P*cad* promoter in the expression vector pCN51, as previously described [3, 6].

### Protein expression and purification

The expression of His-tagged dimeric and trimeric Duts proteins was done in the *E*. *coli* BL21 (DE3) (Novagen) strain transformed with the corresponding gene cloned in pET-28a plasmid (Novagen) (S5 Table) and protein purification was carried out as previously described [6]. For the production of selenomethionine (SeMet) substituted ϕDI Dut used in the SAD experiments, the *E*. *coli* cells were grown in Seleno Met Medium Base plus nutrient mix media (Molecular Dimensions) supplemented with L-Seleno-methionine and kanamycin at 60 and 33 mg/ml final concentrations, respectively. Cells were grown at 37° C at 250 rpm till exponential growth phase (OD_600_ = 0.6). The over-expression of proteins was induced by adding 1 mM Isopropyl-β-D-1-thiogalactopyranoside (IPTG), the temperature was dropped to 20°C and the cells were grown for an additional 16 hours. Protein was purified following the identical protocol as for wild-type Duts. For Stl expression and purification the same protocol as previously described was used [6].

### Native polyacrylamide gel electrophoresis (Native-PAGE)

Native-PAGE was carried out using 8% polyacrylamide gels, running the gels without samples for 30 minutes and afterwards 10 μl of sample was added to each well. Samples were pre-incubated for 20 minutes before adding the loading sample buffer (65.8 mM Tris-HCl pH 6.8, 23,16% (v/v) glycerol and 0.011% (w/v) bromophenol blue) [31]. The Dut proteins were at a 17 μM final concentration and Stl at a 17 or 34 μM. When is indicated nucleotides were added to variable concentrations (from 1 to 1000 μM). The electrophoresis was performed at 4°C, for 3 hours in a 25 mM Tris-HCl, 1.44% (w/v) glycine buffer (pH 8.3). Gels were stained with Coomassie Blue.

### dUTPase and dNTPase activity assay

The dUTPase activity was measured by Malachite Green phosphate assay as previously described [6]. For the dUTPase assay, 1 μg of the corresponding Dut was used, the reactions were started by addition of several dUTP concentrations (from 3 μM up to 400μM), and aliquots were analyzed at different time points (0, 2, 4, 6, 8, and 10 min) measuring the Pi production. The specific activity and the K_M_ for each enzyme were calculated using SigmaPlot software. For the dNTPase activity assay, 2 μg of the corresponding Dut was used and the reactions were started by addition of a final dNTP concentration of 400μM, analyzing the ability to hydrolyze dATP, dCTP, dGTP, dTTP and dITP, using dUTP as a control. Samples were incubated at room temperature for 20 minutes and the P_i_ produce was measured as previously described.

The inhibition of dUTPase activity by Stl was measured following the same procedure, but 30 nM of the analyzed Dut protein was incubated for 16 h at 21°C with incremental concentrations of Stl (0.5, 2 or 10 times molar ratio) prior to starting the reaction by adding 10 μM of dUTP.

### Isothermal Titration Microcalorimetry

Isothermal Titration Microcalorimetry (ITC) was used to calculate the dissociation constant of ϕDI and ϕDI^A73L^ Duts against dUPNPP (2-Deoxyuridine-5-[(α,β)-imido]triphosphate; Jena Biosciences), a nonhydrolyzable dUTP analog. The protein concentration was 20 mM, while the dUPNPP was at 200 mM, diluted in 50 mM HEPES 7.5, 250 mM NaCl, 5mM MgCl_2_. The experiment was performed at 25°C. The ITC experiment was carried out in a Nano ITC Low Volume (TA instruments). The data obtained was integrated, corrected and analyzed using the NanoAnalyze software (TA Instruments) with a single-site binding model.

### Protein crystallization, data collection and structure determination

ϕDI Duts were crystallized at 21°C using sitting drop method in the Crystallogenesis facility of IBV. Proteins were used at 10 mg/mL concentration. To obtain ϕDI Dut crystals in complex with dUPNPP (ϕDI-dUPNPP) the protein was incubated with 1 mM dUPNPP and 5 mM MgCl_2_. Initial crystallization conditions were improved to get crystals that diffracted X-rays at a resolution higher than 3 Å. Final crystallization conditions for each protein were: 28% PEG 6000, 0.5M LiCl_2_ y 0.1M Tris-HCl pH 8.5 for ϕDI *apo* form; 8% PEG 3350, 0.1 M Na-HEPES pH 7.5; 0.2M NaCl, 1.2 M ammonium sulphate and 0.03 M ammonium acetate for ϕDI-dUPNPP; and 30% PEG 400, 0.2 M MgCl_2_, 0.1 M Na-HEPES 7.5 for the ϕDI^A73L^ mutant. Microseeding technique [32] was used to generate crystals of ϕDI-dUPNPP with the proper size and quality for X-ray analysis. Microseeding was performed by mixing 0.4μL of ϕDI-dUPNPP protein solution with 0.3μL crystallization condition and 0.1μL of seeding solution (poor-quality fragmented ϕDI-dUPNPP crystals diluted in crystallization condition). SeMet substituted ϕDI-dUPNPP crystals were as ϕDI-dUPNPP wild-type crystals.

Crystals of ϕDI^A73L^ were directly frozen in liquid nitrogen without any cryobuffer. Crystals of ϕDI *apo* or ϕDI-dUPNPP were frozen using as cryo-protectant the mother liquor conditions increased to 35% PEG 6000 or increased to 16% PEG 3350 and supplemented with 20% sucrose, respectively. X-ray diffraction was performed at 100K in DLS and ALBA synchrotrons.

Processing of collected data was performed with the XDS program [33]. Statistics for processed data are shown in Table 3. Structures were solved at 2.1, 1.85 and 1.90 Å resolution for ϕDI-dUPNPP, ϕDI *apo* and ϕDI^A73L^, respectively. Initial attempts to solve ϕDI-dUPNPP by molecular replacement using PDBs of known dimeric Duts as models failed, suggesting structural differences with these models. Therefore, the structure of ϕDI-dUPNPP was determined by Single-Wavelength Anomalous Dispersion (SAD) using data from SeMet derivative ϕDI-dUPNPP crystals. Autosol pipeline of Phenix [34] was used to process the data and to localize 24 selenium atoms which were enough for calculating experimental phases and to build the initial model to 3.0 Å resolution. Data from native crystals was used to generate the final model at 2.1 Å resolution by interactive cycles of manual model building with Coot [35] and computational refinement with Phenix [34]. Structures for ϕDI *apo* and ϕDI^A73L^ mutant proteins were obtained by molecular replacement using Phaser [36] and the poly-alanine chain of ϕDI-dUPNPP structure excluding residues from 80–150 as starting model. Iterative refinement, rebuilding and validation steps were done using programs Coot and Phenix. Refinement statistics and models composition are shown in Table 3. Stereochemical properties were assessed by wwwPDB X-ray Validation server (https://validate-rcsb-1.wwpdb.org). Superimpositions were calculated using Superpose implemented in the CCP4 suite [37]. Surface accessibility and macromolecular interfaces were calculated using PDBePISA software [38].

### Southern blot sample preparation

Samples were taken at 0 and 90 min following phage/SaPI induction with mitomycin C (Sigma-Aldrich, from *Streptomyces caespitosus*), or at 3 h following plasmid induction with 1–5 μM cadmium (CdCl_2_, Cadmium chloride hemi(pentahydrate), Sigma-Aldrich). Samples were pelleted and frozen at -20°C until all samples were obtained. The samples were re-suspended in 50μl lysis buffer (47.5μl TES-Sucrose and 2.5μl lysostaphin, Sigma-Aldrich from *Staphylococcus staphylolyticus*) and incubated at 37°C for 1 hour. 55μl of SDS 2% proteinase K buffer (47.25μl H2O, 5.25μl SDS 20%, 2.5μl proteinase K, Sigma-Aldrich from *Tritirachium album*) was added before incubation at 55°C for 30 minutes. Samples were vortexed for at least 20 minutes with 11μl of 10X loading dye. Cycles of incubation in dry ice and ethanol, then at 65°C were performed. Samples were run on 0.7% agarose gel at 25V overnight. DNA was transferred to a nylon membrane (0.45 mm hybond-N pore diameter, Amersham Life Science) and exposed using a DIG-labelled probe (Digoxigenin-11-dUTP alkali-labile, Roche) and anti-DIG antibody (Anti-Digoxigenin-AP Fab fragments, Roche) as per the suppliers protocol, before washing and visualisation. The primers used to obtain the labelled probes are shown in S4 Table.

### Nitrocefin assay

For the β-Lactamase assays, cells were obtained at 0.2–0.3 OD_540_ and at 4 and 5 hours post-induction with 5μM CdCl_2_. β-Lactamase assays, using nitrocefin as substrate, were performed as described [2], using a ELx808 microplate reader (BioTek). An adjustment was made in reading time, with plates read every 20 seconds for 30 mins. β-Lactamase units/ml are defined as *[(slope)(Vd)]/[(Em)(l)(s)]*. *Slope* is the Δabsorbance/hour, *V* is the volume of the reaction, *d* is the dilution factor, *Em* is the millimolar extinction coefficient for the nitrocefin (20,500 M^-1^ cm^-1^ at 486 nm), *l* is the path length (cm), and *s* is the sample amount.

#### Western blots

Preparation of *S*. *aureus* samples for western blot was performed by re-suspending pellets in 200μl digestion/lysis buffer (50mM Tris-HCl, 20mM MgCl2, 30% w/v raffinose) plus 1 μl of lysostaphin, mixed briefly, and incubated at 37°C for 1 h. 2X Laemmli sample buffer (Bio-Rad, 2-mercaptoethanol added) was added to the samples, which were heated at 95°C for 10 min, put on ice for 5 min and fast touch centrifuged. Samples were run on SDS-PAGE gels (15% Acrylamide, Bio-Rad 30% Acrylamide/Bis Solution) before transferring to a PVDF transfer membrane (Thermo Scientific, 0.2 μM) using standard methods. Western blot assays were performed using anti-Flag antibody probes (Monoclonal ANTI-FLAG M2-Peroxidase (HRP) antibody produced in mouse, Sigma-Aldrich) as per the protocol supplied by the manufacturer.

## Supporting information

S1 Fig ϕDI Dut represents a reduced version of dimeric Duts. (PDF)

S2 Fig Dimeric Dut mutants do not induce the SaPI cycle. (PDF)

S3 Fig Evaluation of the molar ration in dimeric Dut-Stl interaction. (PDF)

S4 Fig ϕDI Dut in complex with dUPNPP shows a closed conformation. (PDF)

S5 Fig Overlay of ϕDI and *T*. *cruzi* dimer interfaces. (PDF)

S6 Fig IC_50_ calculation of ϕDI-Stl complex formation inhibition by dUPNPP. (PDF)

S7 Fig The A73L mutation in ϕDI Dut prevents dUTP binding. (PDF)

S1 Table Staphylococcal phage dimeric Duts identified by protein BLAST. (PDF)

S2 Table Differences in catalytic motifs between trimeric and dimeric Duts. (PDF)

S3 Table Bacterial strains used in this study. (PDF)

S4 Table Oligonucleotide designs used in this study. (PDF)

S5 Table Plasmids used in this study. (PDF)
