# Supplementary material for: Convergent evolution involving dimeric and trimeric dUTPases in pathogenicity island mobilization
Source: PLoS Pathog. 2017 Sep 11;13(9):e1006581. doi: 10.1371/journal.ppat.1006581 (PMC5608427; doi:10.1371/journal.ppat.1006581)
Supplement: S1 Fig — (PDF) [file ppat.1006581.s001.pdf]

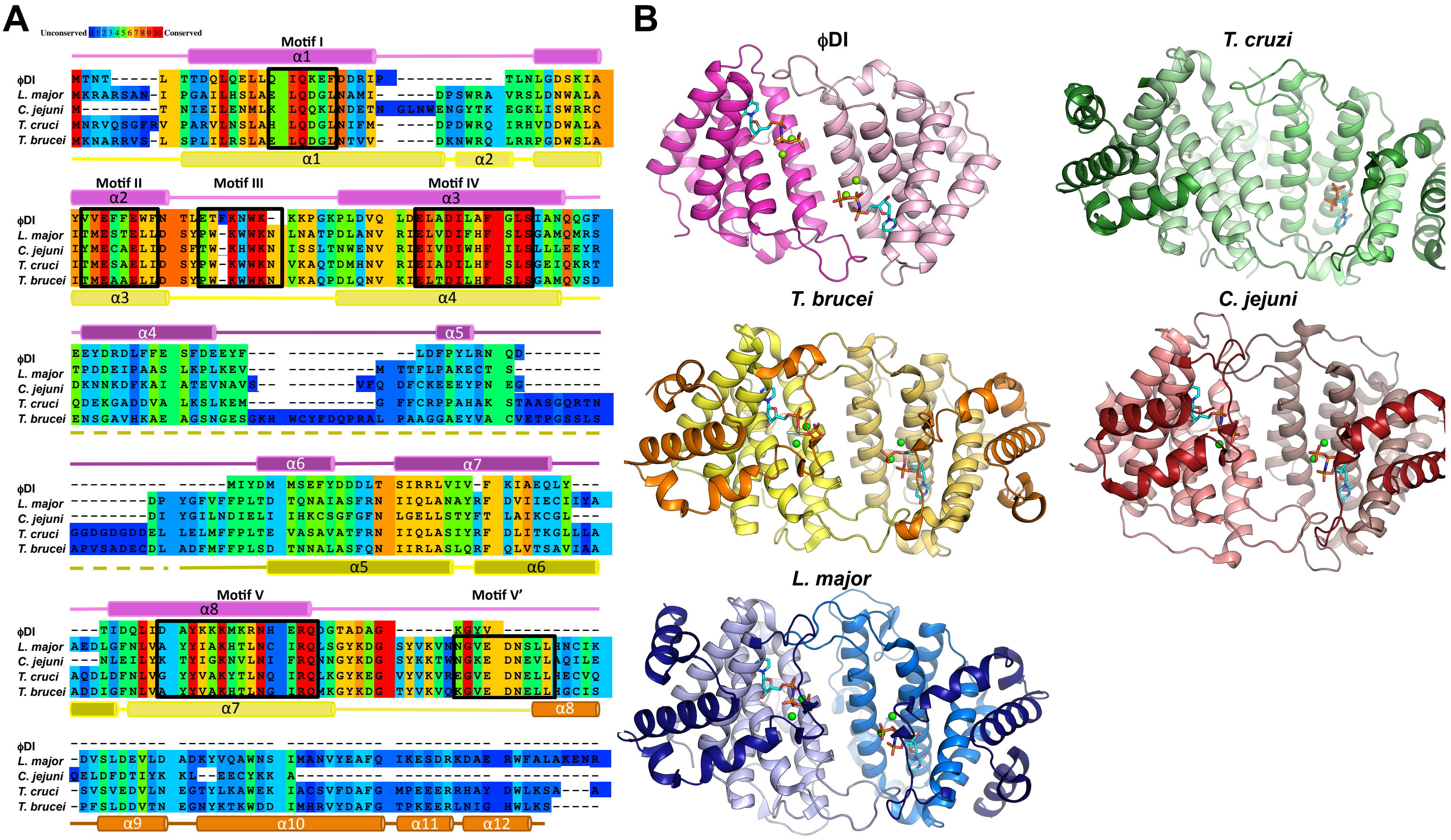

Supplementary Figure 1. ϕDI Dut represents a reduced version of dimeric Duts. (A) Sequence alignment of Duts with known structure. The sequences of the Duts represented in panel (B) are aligned showing the relative sequence conservation at each position, with red being most conserved and blue being least (adapted from alignment generated by PRALINE). The five active site motifs are highlighted in boxes and labeled. The structural elements shown above the alignment correspond to ϕDI and below to *T. brucei* as a representative of the rest of dimeric Duts. The highly variable central motif VI is highlighted with a different tone in the structural elements. In *T. brucei* this region is partly unstructured (denoted by a dashed line). The mobile C-terminal section absent in the ϕDI Dut is highlighted in a darker tone in the structural elements of *T. brucei*, as in panel (B). (B) Known structures of dimeric Duts are shown in cartoon representation and each subunit colored in a tone of magenta for ϕDI, green for *Trypanosoma cruzi* (PDB 1OGK), yellow for *Trypanosoma brucei* (PDB 4DL8), red for *Campylobacter jejuni* (PDB 2CIC) and blue for *Leishmania major* (PDB 2YAY). The mobile C-terminal section, which is absent in the ϕDI Dut, is colored in a darker tone in the rest of Duts.
