# Supplementary material for: Convergent evolution involving dimeric and trimeric dUTPases in pathogenicity island mobilization
Source: PLoS Pathog. 2017 Sep 11;13(9):e1006581. doi: 10.1371/journal.ppat.1006581 (PMC5608427; doi:10.1371/journal.ppat.1006581)
Supplement: S2 Fig — (PDF) [file ppat.1006581.s002.pdf]

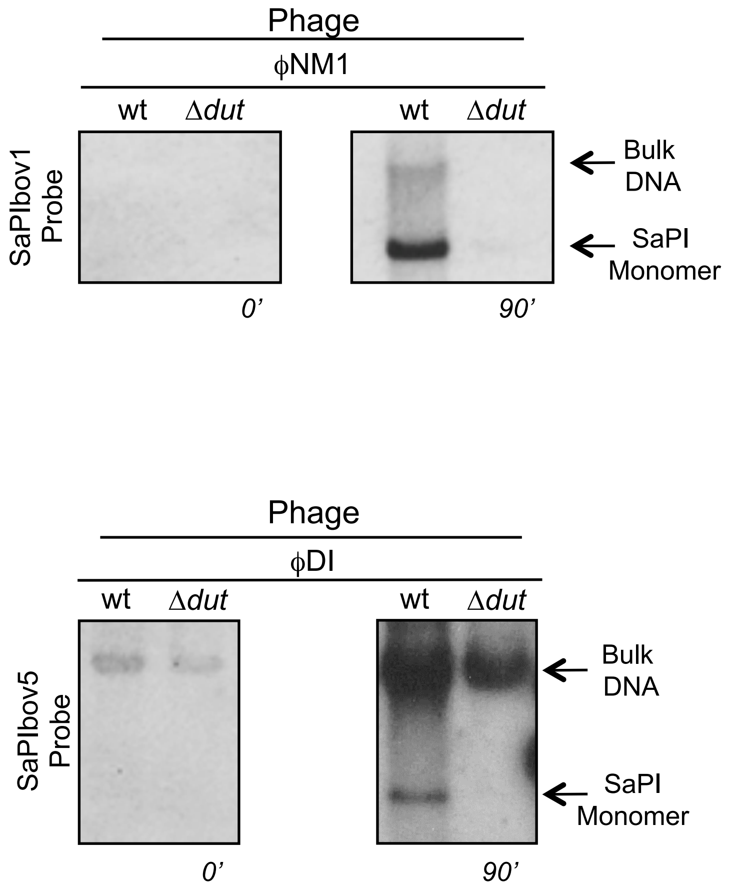

Supplementary Figure 2. Dimeric Dut mutants do not induce the SaPI cycle. SaPIbov1 excision and replication following induction of ϕNM1 and SaPIbov5 replication following induction of ϕDI. Phages either wild-type or mutant in the *dut* gene are shown. Southern blots were performed using strains with SaPIbov1 or SaPIbov5 and respective phages. Samples were isolated at 0’ or 90’ minutes following induction with mitomycin C. Southern blots were performed using a probe for the SaPIbov1/5 integrase. The upper band represents bulk DNA, whilst the lower band signifies the SaPI band
