# Supplementary material for: Convergent evolution involving dimeric and trimeric dUTPases in pathogenicity island mobilization
Source: PLoS Pathog. 2017 Sep 11;13(9):e1006581. doi: 10.1371/journal.ppat.1006581 (PMC5608427; doi:10.1371/journal.ppat.1006581)
Supplement: S3 Fig — (PDF) [file ppat.1006581.s003.pdf]

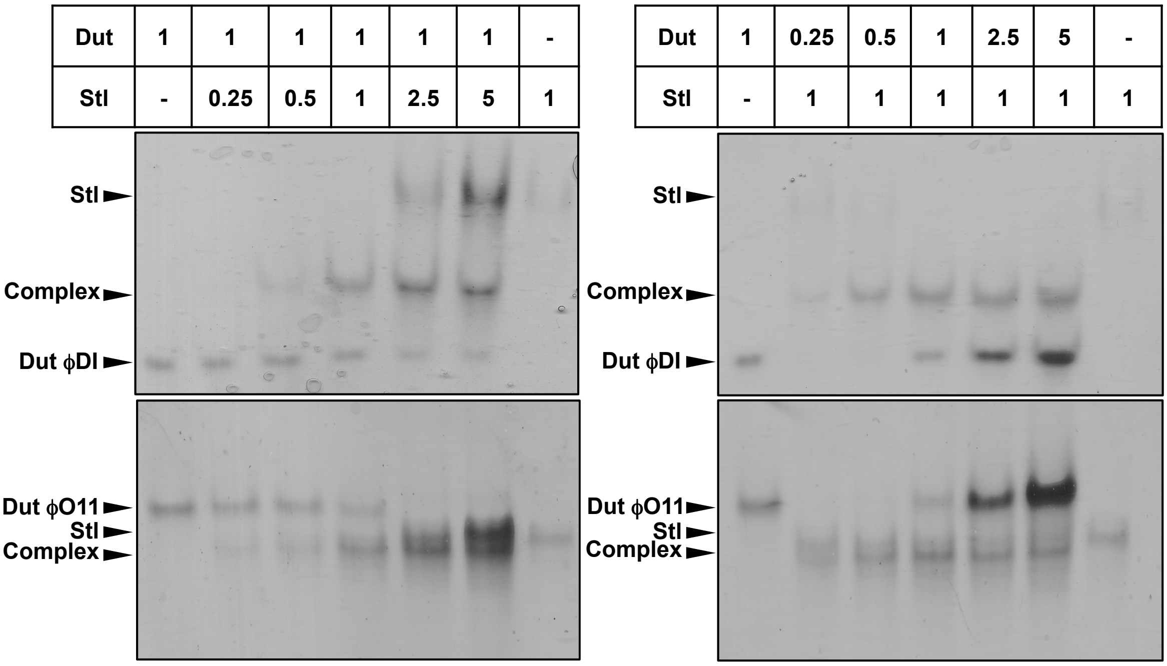

Supplementary Figure 3. Evaluation of the molar ration in dimeric Dut-Stl interaction. (*left panels*) Constant amounts of the ϕDI and ϕO11 inducing dimeric Duts were incubated with variable amounts, from 0.25 to 5 molar times (in monomer), of Stl and the complex formation was evaluated by Native-PAGE. (*right panels*) Inverse titration experiments, maintaining a constant concentration of Stl while varying the amount of Dut. In both titration experiments the 1:1 molar ration generates a band corresponding to the complex with minimum excess of each of the individual proteins, supporting this ratio for the Dut-Stl interaction.
