# Supplementary material for: Convergent evolution involving dimeric and trimeric dUTPases in pathogenicity island mobilization
Source: PLoS Pathog. 2017 Sep 11;13(9):e1006581. doi: 10.1371/journal.ppat.1006581 (PMC5608427; doi:10.1371/journal.ppat.1006581)
Supplement: S4 Fig — (PDF) [file ppat.1006581.s004.pdf]

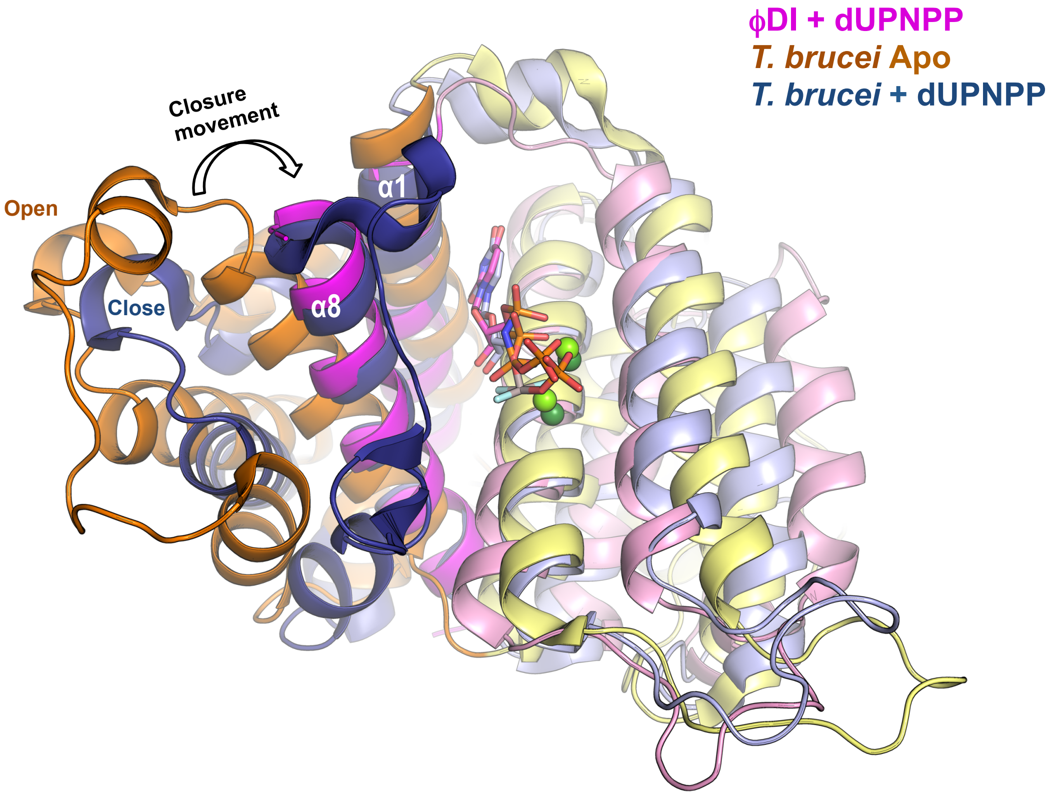


**Supplementary Figure 4. ϕDI Dut in complex with dUPNPP shows a closed conformation.** Cartoon representation of the superimposed protomers of Duts from T. brucei in apo (yellow and orange) and dUPNPP bound (light and dark blue) forms, compared with ϕDI in dUPNPP bound (light and dark pink) form. The *T. brucei* structures exemplify the closing movement induced by the nucleotide that brings the C-terminal mobile part (in dark tones) toward the active center. In the ϕDI structure the mobile portion, which is reduced to helices α1 and α8, shows a closed conformation. Nucleotides from ϕDI- and *T. brucei*–dUPNPP bound structures are represented in sticks with carbon atoms colored according to the protomer to which they correspond.
