# Supplementary material for: Convergent evolution involving dimeric and trimeric dUTPases in pathogenicity island mobilization
Source: PLoS Pathog. 2017 Sep 11;13(9):e1006581. doi: 10.1371/journal.ppat.1006581 (PMC5608427; doi:10.1371/journal.ppat.1006581)
Supplement: S5 Fig — (PDF) [file ppat.1006581.s005.pdf]

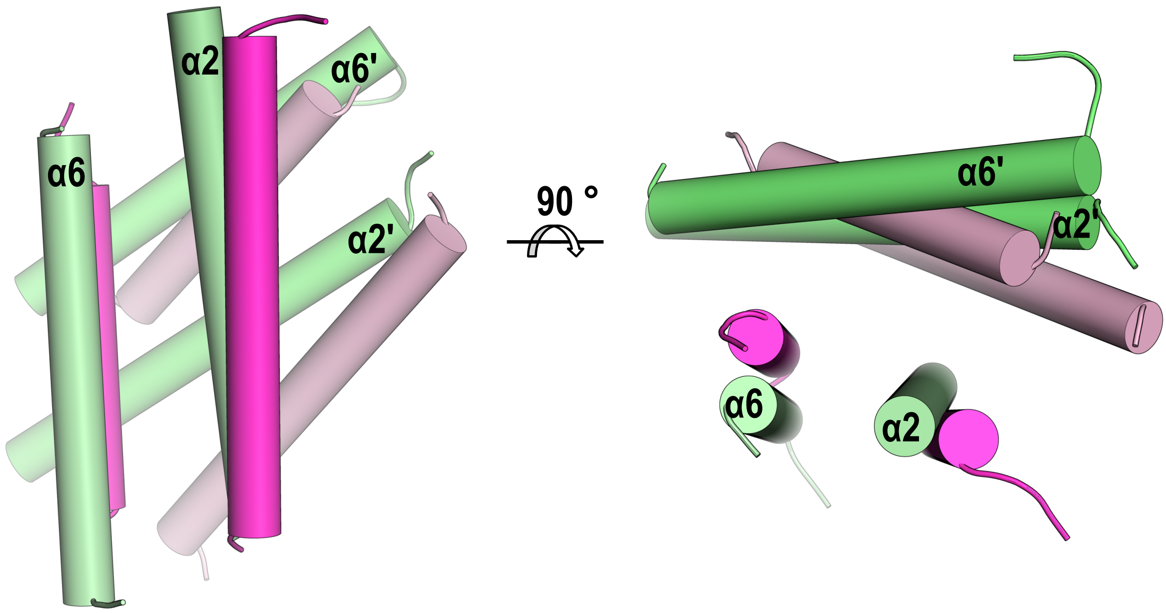

Supplementary Figure 5. Overlay of ϕDI and *T. cruzi* dimer interfaces. Two orthogonal views of the superimposition of helices α2 and α6, the structural elements providing the main part of the dimer interface, from ϕDI (pink tones) and *T. cruzi* (green tones) showing that the α-helices from the *S. aureus* phage Dut presents a more parallel relative orientation. The rearrangement on these α-helices disposition reduces the surface of dimerization.
