# Supplementary material for: Convergent evolution involving dimeric and trimeric dUTPases in pathogenicity island mobilization
Source: PLoS Pathog. 2017 Sep 11;13(9):e1006581. doi: 10.1371/journal.ppat.1006581 (PMC5608427; doi:10.1371/journal.ppat.1006581)
Supplement: S6 Fig — (PDF) [file ppat.1006581.s006.pdf]

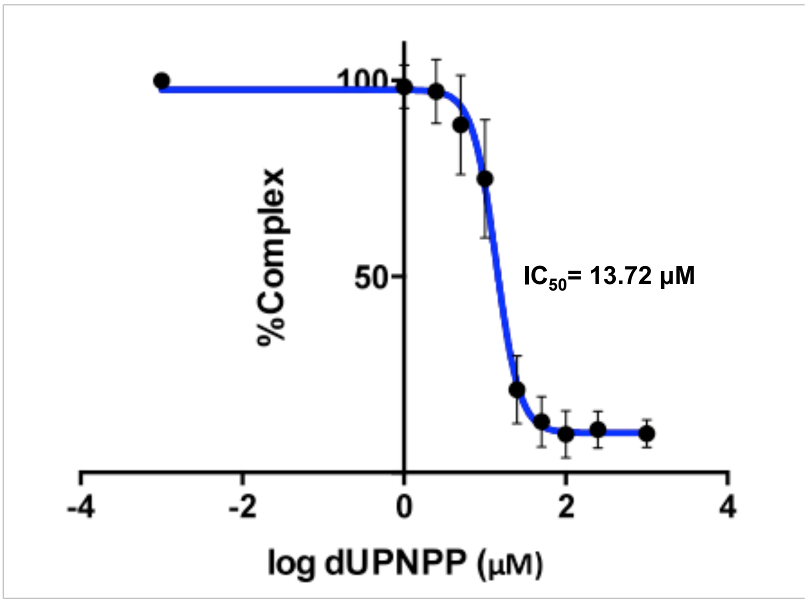

Supplementary Figure 6. IC_{50} calculation of ϕDI-StI complex formation inhibition by dUPNPP. dUPNPP induced inhibition of ϕDI-StI complex formation was evaluated by Native-Page (Fig 5). The bands corresponding to the complex were quantified and normalized corresponding the 100% of complex observed in the absence of dUPNPP. The IC_{50} was calculated by fitting a Hill function to the data. Error bars indicate SDs for 3 independent assays
