# Supplementary material for: Convergent evolution involving dimeric and trimeric dUTPases in pathogenicity island mobilization
Source: PLoS Pathog. 2017 Sep 11;13(9):e1006581. doi: 10.1371/journal.ppat.1006581 (PMC5608427; doi:10.1371/journal.ppat.1006581)
Supplement: S7 Fig — (PDF) [file ppat.1006581.s007.pdf]

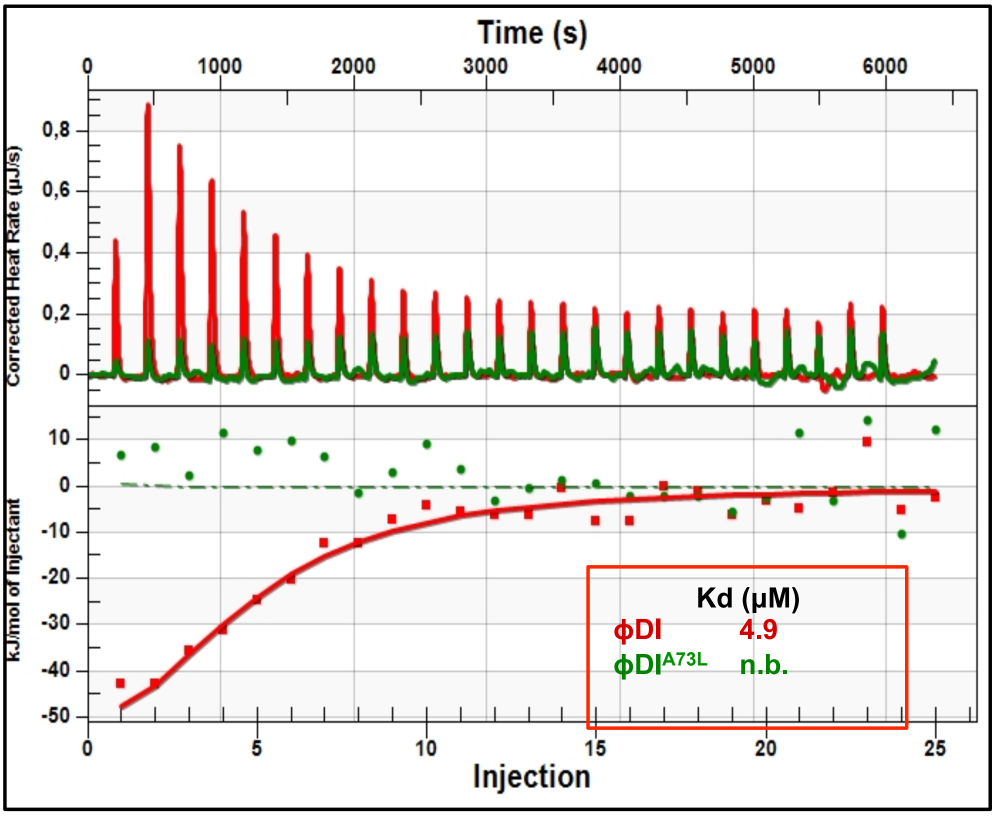

Supplementary Figure 7. The A73L mutation in ϕDI Dut prevents dUTP binding. Nucleotide-binding affinity was measured by ITC for the ϕDI and ϕDI^{A73L} Duts with dUPNPP. The constant *K*d (μM) for the ϕDI wild-type form is shown in the picture. No binding (n.b) was detected for the ϕDI^{A73L} mutant.
