# Supplementary material for: Convergent evolution involving dimeric and trimeric dUTPases in pathogenicity island mobilization
Source: PLoS Pathog. 2017 Sep 11;13(9):e1006581. doi: 10.1371/journal.ppat.1006581 (PMC5608427; doi:10.1371/journal.ppat.1006581)
Supplement: S1 Table — (PDF) [file ppat.1006581.s008.pdf]

**Supplementary Table 1. Staphylococcal phage dimeric Duts identified by protein BLAST.**

| NCBI protein accession number (phage name) | Definition (NCBI) | NCBI organism (assigned) |
|---|---|---|
| YP_873980<br>(ΦNM1 Dut)* | Hypothetical protein SAPPV1_gp31 | Staphylococcus phage phiNM |
| AFO71480 | Putative dUTP diphosphatase | Staphylococcus phage SA13 |
| YP_008239533 | Hypothetical protein JS01_043 | Staphylococcus phage JS01 |
| EFB46834 | Hypothetical protein SASG_02436 | Staphylococcus aureus C427 |
| EFB97382 | Conserved hypothetical protein | Staphylococcus aureus A9765 |
| EHM57234 | dUTP diphosphatase | Staphylococcus aureus 21209 |
| YP_240523<br>(Φ55 Dut)* | ORF023 | Staphylococcus phage 55 |
| ACY11889 | dUTPase | Staphylococcus aureus ED98 |
| ADI96843 | Dimeric dUTPase | Staphylococcus aureus ED133 |
| EFG58024 | Hypothetical protein SIAG_00559 | Staphylococcus aureus EMRSA16 |
| CAG40530 | Hypothetical phage protein | Staphylococcus aureus MRSA252 |
| CAI81421 | Hypothetical phage-related protein | Staphylococcus aureus RF122 |
| EWO63645 | Hypothetical protein Q256_02664 | Staphylococcus aureus M1273 |
| YP_240297 | ORF023 | Staphylococcus phage 96 |
| YP_009204059 | dUTPase | Staphylococcus phage B166 |
| EZU98143 | Hypothetical protein U920_01665 | Staphylococcus aureus 11S00627 |
| EUX77280 | Hypothetical protein O471_02354 | Staphylococcus aureus M0357 |
| Not assigned<br>(ΦDI Dut)* | dUTPase family protein | Staphylococcus phage phiDI |
| CDR62164 | dUTPase family protein | Staphylococcus schweitzeri |
| WP_049431351 | dUTPase | Staphylococcus hominis |
| WP_072098691 | dUTPase | Staphylococcus capitis |
| SAO04495<br>(ΦDII Dut)* | dUTPase family protein | Staphylococcus aureus |
| EZY57710 | Hypothetical protein V060_02804 | Staphylococcus aureus R0294 |
| EZY59344 | Hypothetical protein V061_02764 | Staphylococcus aureus R0353 |
| YP_240735 | ORF025 | Staphylococcus phage 88 |
| YP_240809 | ORF025 | Staphylococcus phage 92 |
| EZY70921 | Hypothetical protein V064_01619 | Staphylococcus aureus R0545 |

| | | |
|---|---|---|
| YP_240887 | ORF023 | Staphylococcus phage X2 |
| YP_002332391 | Putative dUTPase | Staphylococcus phage Ipla35 |
| YP_001949832<br>(ΦMR25 Dut)* | Putative dUTPase | Staphylococcus phage phiMR25 |
| EWR22849 | Phage dUTPase | Staphylococcus aureus M1120 |
| EVP78464 | Phage dUTPase | Staphylococcus aureus M0763 |
| EUR36964 | Phage dUTPase | Staphylococcus aureus GGMC6008 |
| EVJ54045 | Phage dUTPase | Staphylococcus aureus WAMC6080 |
| EWV51383 | Phage dUTPase | Staphylococcus aureus F63687 |
| EYP21990 | Phage dUTPase | Staphylococcus aureus DAR1158 |
| EVM02059 | Phage dUTPase | Staphylococcus aureus M0897 |
| EGA99443<br>(ΦO46 Dut)* | Hypothetical protein SAO46_2267 | Staphylococcus aureus O46 |
| CXE15073 | Dimeric dUTPase | Staphylococcus aureus |
| EZY69567 | Hypothetical protein V065_02754 | Staphylococcus aureus R0611 |
| CXK87292 | Dimeric dUTPase | Staphylococcus aureus |
| EGA96175<br>(ΦO11 Dut) | Hypothetical protein SAO11_2716 | Staphylococcus aureus O11 |
| YP_239632 | ORF026 | Staphylococcus phage 69 |
| SGS40384 | dUTPase family protein | Staphylococcus aureus |
| SAO24456 | dUTPase family protein | Staphylococcus aureus |
| ENL95507 | Phage dUTPase | Staphylococcus aureus M1034 |
| EUR29836 | Phage dUTPase | Staphylococcus aureus SJOS6061 |
| YP_008059848<br>(StauST398-3 Dut)* | hypothetical protein StauST398-3_0032 | Staphylococcus phage StauST398-3 |
| WP_057504496 | dUTPase | Staphylococcus aureus |
| WP_031880022 | dUTPase | Staphylococcus aureus |
| AMO52038 | Dimeric dUTPase | Staphylococcus aureus Tager 104 |
| EHO90986 | dUTP diphosphatase | Staphylococcus aureus 21262 |
| EEV67636 | Phage protein | Staphylococcus aureus A9719 |
| EYN68054 | Phage dUTPase | Staphylococcus aureus DAR3762 |
| EMY95987 | Phage dUTPase | Staphylococcus aureus M0075 |
| ADV05101<br>(HKU10-03 Dut)* | Dimeric dUTPase | Staphylococcus pseudintermedius HKU10-03 |
| ANQ81944 | dUTPase | Staphylococcus pseudintermedius |

| | | |
|---|---|---|
| SGS40384 | dUTPase family protein | Staphylococcus aureus |
| OAW38013 | dUTPase | Staphylococcus epidermidis |
| **Trimeric dUTPases (outliers)** | | |
| **NCBI protein accession number (phage name)** | **Definition (NCBI)** | **NCBI organism (assigned)** |
| WP_011447036 (Φ11) | dUTP pyrophosphatase | Staphylococcus aureus |
| YP_001285346 (80α) | dUTPase | Staphylococcus phage 80alpha |
| AAL82303 (Φ12/Φ85) | dUTPase | Staphylococcus phage Phi12 |
| NP_075491 (ΦSLT) | phi PVL ORF 53 analogue | Staphylococcus phage Slt |

*Representatives of the 9 different families identified by neighbour joining an alignment of all strains tabled here.
