# Supplementary material for: Convergent evolution involving dimeric and trimeric dUTPases in pathogenicity island mobilization
Source: PLoS Pathog. 2017 Sep 11;13(9):e1006581. doi: 10.1371/journal.ppat.1006581 (PMC5608427; doi:10.1371/journal.ppat.1006581)
Supplement: S2 Table — (PDF) [file ppat.1006581.s009.pdf]

Supplementary Table 2. Differences in catalytic motifs between trimeric and dimeric Duts.

	Catalytic motifs[a]				
	I	II	III	IV	V
Dimeric Duts	QIQKEF[b]	VVEFFEWF	ETFKNWK	ELADMLAFGLS	DAYKKKMKRNHERQD
Trimeric Duts	AGYDIFSA	RSGVS	GKIDAGYHG	GDKLAQ	RGEKGFGSSG

[a]Consensus sequence in catalytic motifs in Duts from *S. aureus* phages
[b]Residues in black and grey are completely or partialy conserved in all the sequences analyzed
