# Supplementary material for: Convergent evolution involving dimeric and trimeric dUTPases in pathogenicity island mobilization
Source: PLoS Pathog. 2017 Sep 11;13(9):e1006581. doi: 10.1371/journal.ppat.1006581 (PMC5608427; doi:10.1371/journal.ppat.1006581)
Supplement: S3 Table — (PDF) [file ppat.1006581.s010.pdf]

Supplementary Table 3. Bacterial strains used in this study.

Strains	Description	Reference
DH5α	Host for DNA cloning	
RN4220	Restriction-defective derivate of RN450	(Kreiswirth et al., 1983)
BL21 (DE3)	*E. coli* expression strain	Stratagene
JP6774	RN4220 Δ*spa* SaPIbov1 *tst*::*tet*M	(Tormo-Más et al., 2010)
JP11634	RN4220 SaPIbov5::*erm*C adjusted	(Carpena et al., 2016)
JP16140	RN4220 Δ*spa* SaPIbov5 *vwm::tetM*	This work
JP12491	RN4220 pJP674	(Tormo-Más et al., 2010)
JP10280	JP6774 pJP1927	This work
JP14649	JP6774 pJP1928	This work
JP11742	JP6774 pJP2040	This work
JP14670	JP6774 pJP2041	This work
JP14834	JP6774 pJP2042	This work
JP10545	JP6774 pJP2043	This work
JP12276	JP6774 pJP2044	This work
JP15291	JP6774 pCN51	This work
JP13056	JP12491 pJP1927	This work
JP14809	JP12491 pJP1928	This work
JP13058	JP12491 pJP2040	This work
JP14808	JP12491 pJP2041	This work
JP15292	JP12491 pJP2042	This work
JP13055	JP12491 pJP2043	This work
JP15105	RN4220 pJP674 pCN51	This work
JP13077	JP6774 $\Phi NM1^{WT}$	This work
JP13078	JP6774 $\Phi NM1^{\Delta Dut}$	This work
JP12698	JP11634 ΦDI^{WT}	This work
JP12645	JP11634 $\Phi DI^{\Delta Dut}$	This work
	BL21 (DE3) pJP2045	This work
JP10558	BL21 (DE3) pJP2046	This work
JP10559	BL21 (DE3) pJP2047	This work
JP10560	BL21 (DE3) pJP2048	This work
	BL21 (DE3) pJP2049	This work
JP16197	JP16140 pJP1927	This work
JP16198	JP16140 pJP1928	This work
JP16199	JP16140 pJP2040	This work
JP16200	JP16140 pJP2041	This work
JP16201	JP16140 pJP2042	This work
JP16202	JP16140 pJP2043	This work
JP16203	JP16140 pCN51	This work
**Strains**	**Description**	**Reference**

DH5α	Host for DNA cloning	
RN4220	Restriction-defective derivate of RN450	(Kreiswirth et al., 1983)
BL21 (DE3)	*E. coli* expression strain	Stratagene
JP6774	RN4220 Δ*spa* SaPIbov1 *tst*::*tet*M	(Tormo-Más et al., 2010)
JP11634	RN4220 SaPIbov5::*erm*C adjusted	(Carpena et al., 2016)
JP12491	RN4220 pJP674	(Tormo-Más et al., 2010)
JP10280	JP6774 pJP1927	This work
JP14649	JP6774 pJP1928	This work
JP11742	JP6774 pJP2040	This work
JP14670	JP6774 pJP2041	This work
JP14834	JP6774 pJP2042	This work
JP10545	JP6774 pJP2043	This work
JP12276	JP6774 pJP2044	This work
JP15291	JP6774 pCN51	This work
JP13056	JP12491 pJP1927	This work
JP14809	JP12491 pJP1928	This work
JP13058	JP12491 pJP2040	This work
JP14808	JP12491 pJP2041	This work
JP15292	JP12491 pJP2042	This work
JP13055	JP12491 pJP2043	This work
JP15105	RN4220 pJP674 pCN51	This work
JP13077	JP6774 $\Phi NM1^{WT}$	This work
JP13078	JP6774 $\Phi NM1^{\Delta Dut}$	This work
JP12698	JP11634 ΦDI^{WT}	This work
JP12645	JP11634 $\Phi DI^{\Delta Dut}$	This work
JP13198	JP11634 $\Phi DI^{3xFLAG\text{-}WT\text{-}Dut}$	This work
JP13222	JP11634 $\Phi DI^{3xFLAG\text{-}A73L\text{-}Dut}$	This work

References

Carpena, N., Manning, K.A., Dokland, T., Marina, A., and Penadés, J.R. (2016). Convergent evolution of pathogenicity islands in helper cos phage interference. Philos. Trans. R. Soc. Lond., B, Biol. Sci. *371*, 20150505.

Charpentier, E., Anton, A.I., Barry, P., Alfonso, B., Fang, Y., and Novick, R.P. (2004). Novel cassette-based shuttle vector system for gram-positive bacteria. Appl. Environ. Microbiol. *70*, 6076–6085.

Kreiswirth, B.N., Löfdahl, S., Betley, M.J., O'Reilly, M., Schlievert, P.M., Bergdoll, M.S., and Novick, R.P. (1983). The toxic shock syndrome exotoxin structural gene is not detectably transmitted by a prophage. Nature *305*, 709–712.

Tormo-Más, M.Á., Mir, I., Shrestha, A., Tallent, S.M., Campoy, S., Lasa, I., Barbé, J., Novick, R.P., Christie, G.E., and Penadés, J.R. (2010). Moonlighting bacteriophage proteins derepress staphylococcal pathogenicity islands. Nature *465*, 779–782.
