# Supplementary material for: Convergent evolution involving dimeric and trimeric dUTPases in pathogenicity island mobilization
Source: PLoS Pathog. 2017 Sep 11;13(9):e1006581. doi: 10.1371/journal.ppat.1006581 (PMC5608427; doi:10.1371/journal.ppat.1006581)
Supplement: S4 Table — (PDF) [file ppat.1006581.s011.pdf]

Supplementary Table 4. Oligonucleotide designs used in this study.

Plasmid	Oligonucleotides	Sequence
pJP674	SaPIbov1-149cB<br>NY-24mK	CGCGGATCCGATCAGTACCTAAATATGCG<br>CGGGGTACCCACTCGGTTATAACCTT
pJP1928<br>pJP1927<br>pJP2040<br>pJP2041	dutNM1-1mS	ACGCGTCGACATTATGACGGGTCAAGTTGTCTATAA<br>ATATGAGGAGGCACAGGAAAATGGATTATAAAGATC<br>ACGATGGCGATTATAAAGATC
	dutNM1-2m	CACGATGGCGATTATAAAGATCACGATATCGATTAT<br>AAAGATGATGATGATAAAATGACTAACACATTAACA<br>ATTGATCAG
	dut-DI-2cB	CGCGGATCCTTACACGTATCCTTTTCCTGC<br><br>(These plasmids were constructed using the same primers with different templates.)
pJP2042	dutNM1-1mS	ACGCGTCGACATTATGACGGGTCAAGTTGTCTATAA<br>ATATGAGGAGGCACAGGAAAATGGATTATAAAGATC<br>ACGATGGCGATTATAAAGATC
	dutNM1-2m	CACGATGGCGATTATAAAGATCACGATATCGATTAT<br>AAAGATGATGATGATAAAATGACTAACACATTAACA<br>ATTGATCAG
	dutO46-4cB	CGCGGATCCTTACACGTATCCTTTTCCTG
pJP2043	dutNM1-1mS	ACGCGTCGACATTATGACGGGTCAAGTTGTCTATAA<br>ATATGAGGAGGCACAGGAAAATGGATTATAAAGATC<br>ACGATGGCGATTATAAAGATC
	phi55-dut-22m	CACGATGGCGATTATAAAGATCACGATATCGATTAT<br>AAAGATGATGATGATAAAATGACTAACACATTAACA<br>ATTGATC
	dut-DI-2cB	CGCGGATCCTTACACGTATCCTTTTCCTGC
pJP2044	dutNM1-1mS	ACGCGTCGACATTATGACGGGTCAAGTTGTCTATAA<br>ATATGAGGAGGCACAGGAAAATGGATTATAAAGATC<br>ACGATGGCGATTATAAAGATC

	DI-dut-4c	GATTTGCAATACTCAGTCCAAATAATAAGATATCCG CCAATTCATCTAACTG
	DI-dut-3m	CAGTTAGATGAATTGGCGGATATCTTATTATTTGGA CTGAGTATTGCAAATC
	dut-DI-2cB	CGCGGATCCTTACACGTATCCTTTTCCTGC
pJP2045<br>pJP2046<br>pJP2048<br>pJP2049	DI-dut-29mB<br>dutNM1-19cS	CGCGGATCCATGACTAACACATTAACAACTGATCAG<br>ACGCGTCGACTTACACGTATCCTTTTCCTGC<br>(These plasmids were constructed using the same primers with different templates.)
pJP2047	dut-DII-4mB<br>dut NM1-19cS	CGCGGATCCATGACTAACACGTTAACAATTGATCAG<br>ACGCGTCGACTTACACGTATCCTTTTCCTGC

Sequences recognized by the restriction enzymes used in cloning are underlined.

Probe	Oligonucleotides	Sequence
**SaPIbov1/<br>SaPIbov5**	SaPIbov1-112mE<br>SaPIbov1-113cB	CCGGAATTCAATTGCTGAGGCAAAACTTC<br>CGCGGATCCTAATTCTCCACGTCTAAAGC
