# Supplementary material for: Convergent evolution involving dimeric and trimeric dUTPases in pathogenicity island mobilization
Source: PLoS Pathog. 2017 Sep 11;13(9):e1006581. doi: 10.1371/journal.ppat.1006581 (PMC5608427; doi:10.1371/journal.ppat.1006581)
Supplement: S5 Table — (PDF) [file ppat.1006581.s012.pdf]

Supplementary Table 5. Plasmids used in this study.

Plasmid	Description	Reference
pPROEX HTa	Expression vector	Invitrogen
pCN41	Expression vector	(Charpentier et al., 2004)
pCN51	Expression vector	(Charpentier et al., 2004)
pJP674	pRN8298-chlor-pInt-20-19-18blaZ (SaPIbov1)	(Tormo-Más et al., 2010)
pJP1927	pCN51 3xflag ΦNM1 Dut^{WT}	This work
pJP1928	pCN51 3xflag ΦO11 Dut^{WT}	This work
pJP2040	pCN51 3xflag ΦDI Dut^{WT}	This work
pJP2041	pCN51 3xflag ΦDII Dut^{WT}	This work
pJP2042	pCN51 3xflag ΦO46 Dut^{WT}	This work
pJP2043	pCN51 3xflag Φ55 Dut^{WT}	This work
pJP2044	pCN51 3xflag ΦDI Dut^{A73L}	This work
pJP2045	pET-28a ΦDI Dut^{A73L}	This work
pJP2046	pET-28a ΦDI Dut^{WT}	This work
pJP2047	pET-28a ΦDII Dut^{WT}	This work
pJP2048	pET-28a ΦO11 Dut^{WT}	This work
pJP2049	pET-28a Φ55 Dut^{WT}	This work

References

Carpena, N., Manning, K.A., Dokland, T., Marina, A., and Penadés, J.R. (2016). Convergent evolution of pathogenicity islands in helper cos phage interference. Philos. Trans. R. Soc. Lond., B, Biol. Sci. *371*, 20150505.

Charpentier, E., Anton, A.I., Barry, P., Alfonso, B., Fang, Y., and Novick, R.P. (2004). Novel cassette-based shuttle vector system for gram-positive bacteria. Appl. Environ. Microbiol. *70*, 6076–6085.

Kreiswirth, B.N., Löfdahl, S., Betley, M.J., O'Reilly, M., Schlievert, P.M., Bergdoll, M.S., and Novick, R.P. (1983). The toxic shock syndrome exotoxin structural gene is not detectably transmitted by a prophage. Nature *305*, 709–712.

Tormo-Más, M.Á., Mir, I., Shrestha, A., Tallent, S.M., Campoy, S., Lasa, I., Barbé, J., Novick, R.P., Christie, G.E., and Penadés, J.R. (2010). Moonlighting bacteriophage proteins derepress staphylococcal pathogenicity islands. Nature *465*, 779–782.
